# DSCF-DET: An RT-DETR-Based Framework for Fine-Grained Detection of Musk Deer and Visually Similar Artiodactyls

**DOI:** 10.3390/biology15161344

**Published:** 2026-08-08

**Authors:** Jingwen Ji, Yan Wang, Yuhao Zhang, Xianpei Zhu, Kaiwen Guo, Xiaodong Sun, Qin Chen, Bing Niu

**Affiliations:** 1 School of Life Sciences, Shanghai University, Shanghai 200444, China; jingwenji@shu.edu.cn (J.J.); 1982151905@shu.edu.cn (Y.Z.); zhuxianpei@shu.edu.cn (X.Z.); guokw@shu.edu.cn (K.G.); 2 Shanghai Customs, Shanghai 200135, China; wangyan311821@sina.com; 3 School of Medicine, Shanghai University, Shanghai 200444, China; sxd111111@shu.edu.cn

**Keywords:** musk deer detection, wildlife conservation, RT-DETR, object detection, feature fusion, ecological monitoring

## Abstract

Musk deer are forest-dwelling artiodactyls of high conservation value, but their wild populations remain under severe conservation pressure due to poaching driven by the demand for natural musk, together with habitat fragmentation and habitat loss. Efficient non-invasive image-based monitoring is therefore important for musk deer conservation; however, fine-grained detection of musk deer and visually similar artiodactyls in ecological images remains difficult because of background camouflage, vegetation occlusion, and high inter-class similarity. In this study, a fine-grained wildlife image dataset was constructed, and an RT-DETR-based framework, termed DSCF-DET, was proposed for automated detection in complex natural scenes. DSCF-DET integrates three task-oriented modules: DRPBlock for receptive-field-aware feature extraction, SASTE for sparse spatial encoding, and CBAFusion for cross-level feature fusion. On the constructed dataset, DSCF-DET achieved 91.4% precision, 86.2% recall, 88.7% F1-score, and 86.4% mAP50. Compared with RT-DETR-r18, it improved these metrics by 8.6, 12.1, 10.5, and 12.4 percentage points, respectively, while maintaining moderate model complexity. Visualization results showed more target-focused feature responses and reduced background-related activations. Cross-dataset experiments on an independent public wildlife dataset further suggested potential applicability to broader wildlife detection scenarios. These results indicate that DSCF-DET provides a computationally balanced approach for ecological image screening and intelligent musk deer monitoring.

## Simple Summary

Musk deer are valuable forest-dwelling animals, with an estimated global wild population of no more than approximately 300,000 individuals. Their wild populations are threatened by illegal hunting for natural musk and by habitat loss. Accurate monitoring of musk deer is important for conservation, yet identifying them from large numbers of ecological images is difficult because they often live in complex habitats and can look similar to other artiodactyl species (*Elaphodus cephalophus*, *Naemorhedus goral*, *Hydropotes inermis*, *Naemorhedus griseus*, and *Capreolus pygargus*). In this study, we developed an automated image detection method for musk deer and visually similar artiodactyls. The method was designed to improve recognition when animals are partly hidden by vegetation, blend into the background, or show only subtle visual differences from related species. Experiments on our fine-grained wildlife image dataset showed that the proposed method improved detection performance compared with the original model. Visual results also showed that the model focused more clearly on animal body regions and was less affected by background interference. Additional testing on an independent public wildlife dataset further showed that the method may be useful in broader wildlife detection scenarios. This study provides a practical tool for reducing manual image screening work and supporting intelligent musk deer monitoring.

## 1. Introduction

The genus *Moschus* belongs to the infraorder Pecora within the order Artiodactyla and represents a group of small forest-dwelling artiodactyls with high conservation value. In China, the recognized species within this genus currently include *M. berezovskii*, *M. anhuiensis*, *M. chrysogaster*, *M. fuscus*, *M. leucogaster*, and *M. moschiferus* [1]. Musk deer are of particular conservation concern because adult males are targeted by poachers for natural musk. Natural musk has long been used in traditional Chinese medicine and is characterized by high medicinal and economic value [2,3,4]. However, the high value of natural musk has driven its market price to extremely high levels, in some cases reaching several times the value of gold [5,6]. Recent global synthesis estimated the total wild population of musk deer to be no more than approximately 300,000 individuals worldwide and highlighted poaching pressure, habitat fragmentation and habitat loss as continuing threats [7].

Close attention has been paid to methods and techniques for wildlife identification and monitoring [8,9]. However, field identification and monitoring of musk deer remain challenging. Traditional surveys primarily rely on external morphological characteristics, such as body size, coat color, body contour, and posture. However, accurate identification is often difficult in complex natural environments, particularly for non-specialists. Musk deer frequently coexist with morphologically similar artiodactyls, including *Elaphodus cephalophus* (Milne-Edwards, 1871), *Naemorhedus goral* (Hardwicke, 1825), *Hydropotes inermis* (Swinhoe, 1870), *Naemorhedus griseus* (Milne-Edwards, 1871) and *Capreolus pygargus* (Pallas, 1771), which may exhibit similar body shapes, fur textures, and behavioral postures in field images. Molecular approaches, such as mitochondrial genomic analysis, can provide reliable species identification and population genetic information [10,11,12]. Nevertheless, these methods generally require biological samples, laboratory facilities, specialized reagents, and relatively long processing times, limiting their suitability for rapid field screening and real-time ecological monitoring. Therefore, efficient, accurate, and non-invasive identification approaches are needed to obtain timely information on musk deer distribution and ecological behavior.

Image-based monitoring techniques, including camera traps, field photography, and unmanned aerial vehicle (UAV) surveys, have become important tools for wildlife conservation and ecological monitoring [13,14]. These methods can continuously record species occurrence, activity patterns, and habitat use while minimizing disturbance to target animals. However, they also generate large volumes of wildlife monitoring images that require efficient screening and identification. In long-term biodiversity monitoring programs, manual image processing is labor-intensive, time-consuming, and susceptible to observer-dependent variability, particularly when image quality is compromised by complex environmental conditions [15,16]. Recent advances in deep learning have significantly improved automated wildlife detection and ecological image analysis [17,18]. Convolutional neural network (CNN)-based detectors and transformer-based object detection frameworks have demonstrated promising performance in camera-trap image processing, species recognition, and biodiversity monitoring [19,20,21,22]. Nevertheless, the reliable detection of musk deer in natural environments remains a challenging task. Musk deer often inhabit vegetation-dominated, rocky, or shadowed habitats, where their body color and texture closely resemble the surrounding background. Furthermore, partial occlusion caused by vegetation and terrain, together with subtle morphological differences from visually similar artiodactyls, substantially increase the risks of missed detections, false positives, and species-level confusion [23,24,25]. Although existing deep learning models have achieved considerable success in wildlife monitoring, their ability to discriminate among morphologically similar species is often limited, and their performance may deteriorate under camouflage and occlusion conditions. These limitations highlight the need for a robust and computationally balanced framework capable of accurate fine-grained wildlife recognition under complex ecological conditions.

To address these challenges, this study aimed to develop and systematically assess DSCF-DET, an RT-DETR-based framework for the fine-grained detection of musk deer and visually similar artiodactyls in complex ecological images. A dedicated wildlife image dataset was constructed to support model development and evaluation under conditions involving camouflage, vegetation occlusion, complex backgrounds, and high inter-class similarity. Within the RT-DETR framework, DRPBlock, SASTE, and CBAFusion were introduced to improve contextual feature extraction, reduce redundant interactions with background regions, and strengthen the representation of discriminative features across multiple scales. The framework was assessed through comparisons with representative object detectors, module-level ablation experiments, feature visualization, and testing on an independent public wildlife dataset. Beyond automated species identification, this study sought to support the screening of ecological images collected by camera traps, fixed monitoring devices, and UAV surveys, particularly in remote mountainous and forest habitats where repeated field surveys are difficult. More efficient identification of musk deer in these images may help expand monitoring coverage, reduce the burden of manual image review, and provide supporting information for species occurrence assessment, population monitoring, habitat management, conservation patrol planning, and anti-poaching surveillance. Overall, this study aimed to provide a practical technical basis for intelligent musk deer monitoring and broader biodiversity conservation.

## 2. Materials and Methods

### 2.1. Fine-Grained Wildlife Dataset

**Figure 1:**
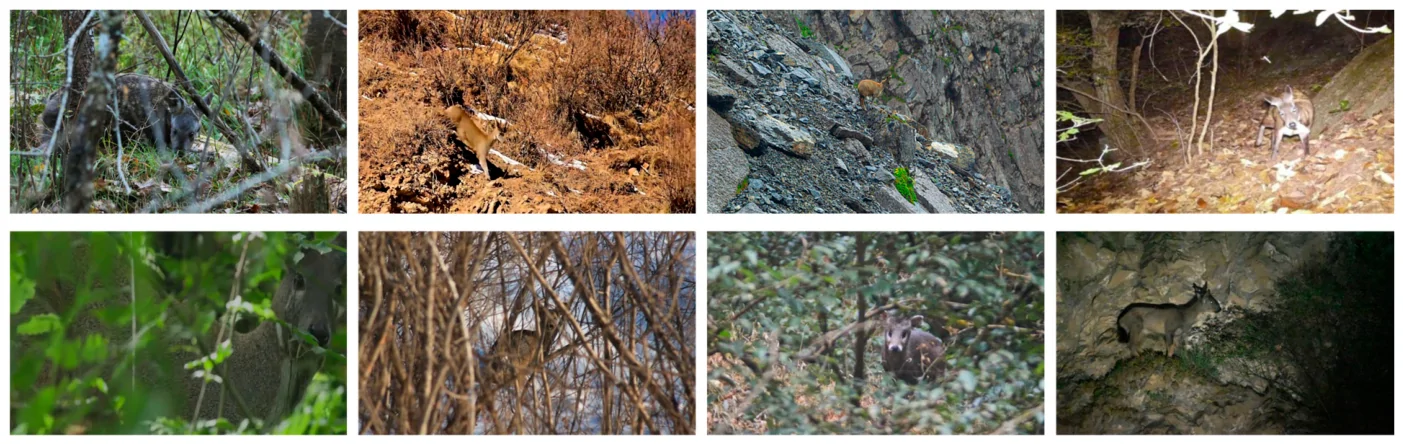
Representative wildlife samples showing camouflage, vegetation occlusion, cluttered natural backgrounds, and nighttime imaging conditions.

To investigate fine-grained wildlife detection in complex natural scenes, a dedicated wildlife dataset was constructed using multi-source ecological image resources. The images were collected from multiple ecological image resources, primarily iNaturalist, https://www.inaturalist.org/ (accessed on 31 March 2025) and Nature Picture Library, https://www.naturepl.com/ (accessed on 31 March 2025), with additional frames extracted from the Chinese wildlife documentary *MijingZhiyan*. Unlike conventional wildlife datasets that mainly contain salient animal targets under relatively clear backgrounds, the proposed dataset was designed to emphasize challenging detection scenarios commonly encountered in real field environments, including background camouflage, vegetation occlusion, inter-class morphological similarity, and illumination variation across daytime and nighttime images (Figure 1).

The dataset contains multiple visually similar artiodactyl species, including *M. fuscus*, *M. chrysogaster*, *M. moschiferus*, *M. berezovskii*, *M. anhuiensis*, *E. cephalophus*, *N. griseus*, *N. goral*, *H. inermis*, and *C. pygargus* (Table 1). Additional characteristics of the dataset are provided in the Supplementary Materials, including the number of annotated objects per species (Table S1), the normalized object-size distribution (Figure S1), the original image-resolution distribution (Figure S2), and the proportions of daytime, nighttime/twilight, occluded, and camouflaged images (Table S2). These species form a representative benchmark for evaluating fine-grained wildlife detection under complex environmental conditions.

**Figure 2:**
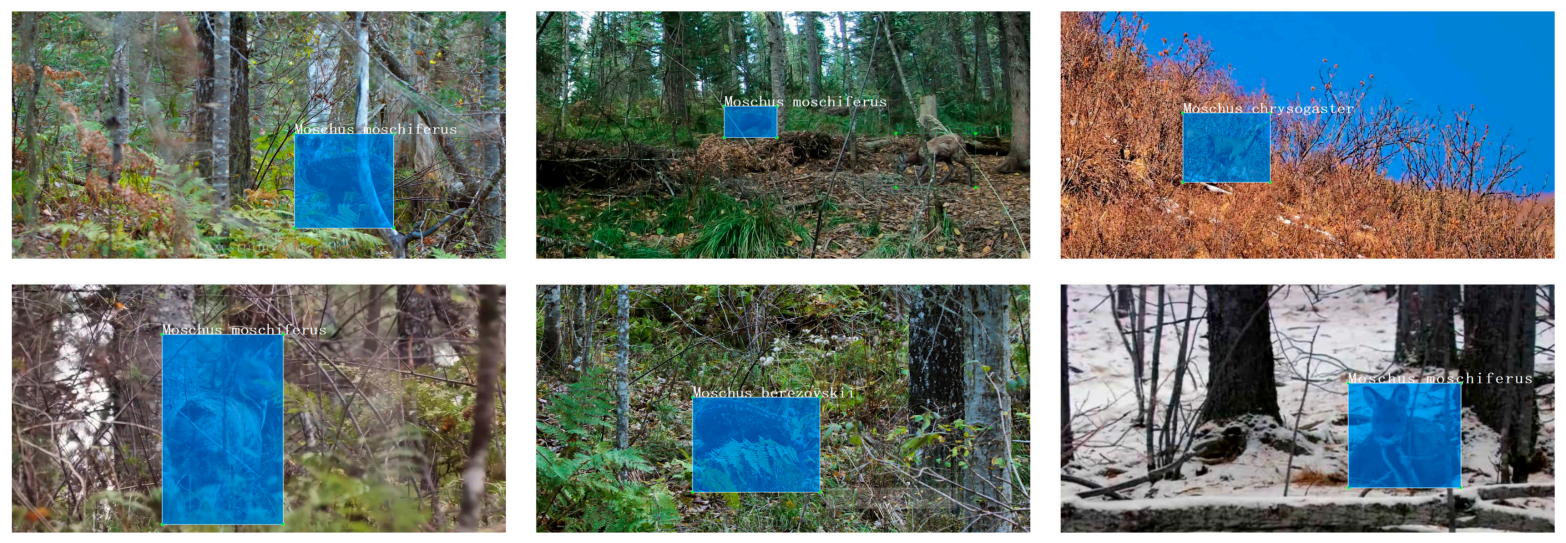
Annotation examples with bounding boxes.

To improve data quality and reduce annotation noise, duplicate, low-resolution, severely blurred, invalid, and taxonomically ambiguous images were removed during preprocessing. All animal instances were manually annotated using labelImg v1.8.6 by three experts with relevant backgrounds in biology and wildlife identification. Species labels were assigned according to the species information accompanying the original records from iNaturalist and Nature Picture Library and, for frames derived from the *MijingZhiyan* documentary materials, explicit species identification or supporting contextual information in the documentary. Images for which species identity could not be reliably confirmed were excluded. Bounding boxes were drawn to cover the visible animal targets as completely as possible while minimizing the inclusion of surrounding background regions, and the finalized annotations were stored in YOLO format (Figure 2). After the initial annotation, other members of the research group conducted an independent quality check of species-label consistency, bounding-box completeness, missing or duplicated annotations, and correspondence between images and annotation files.

**Figure 3:**
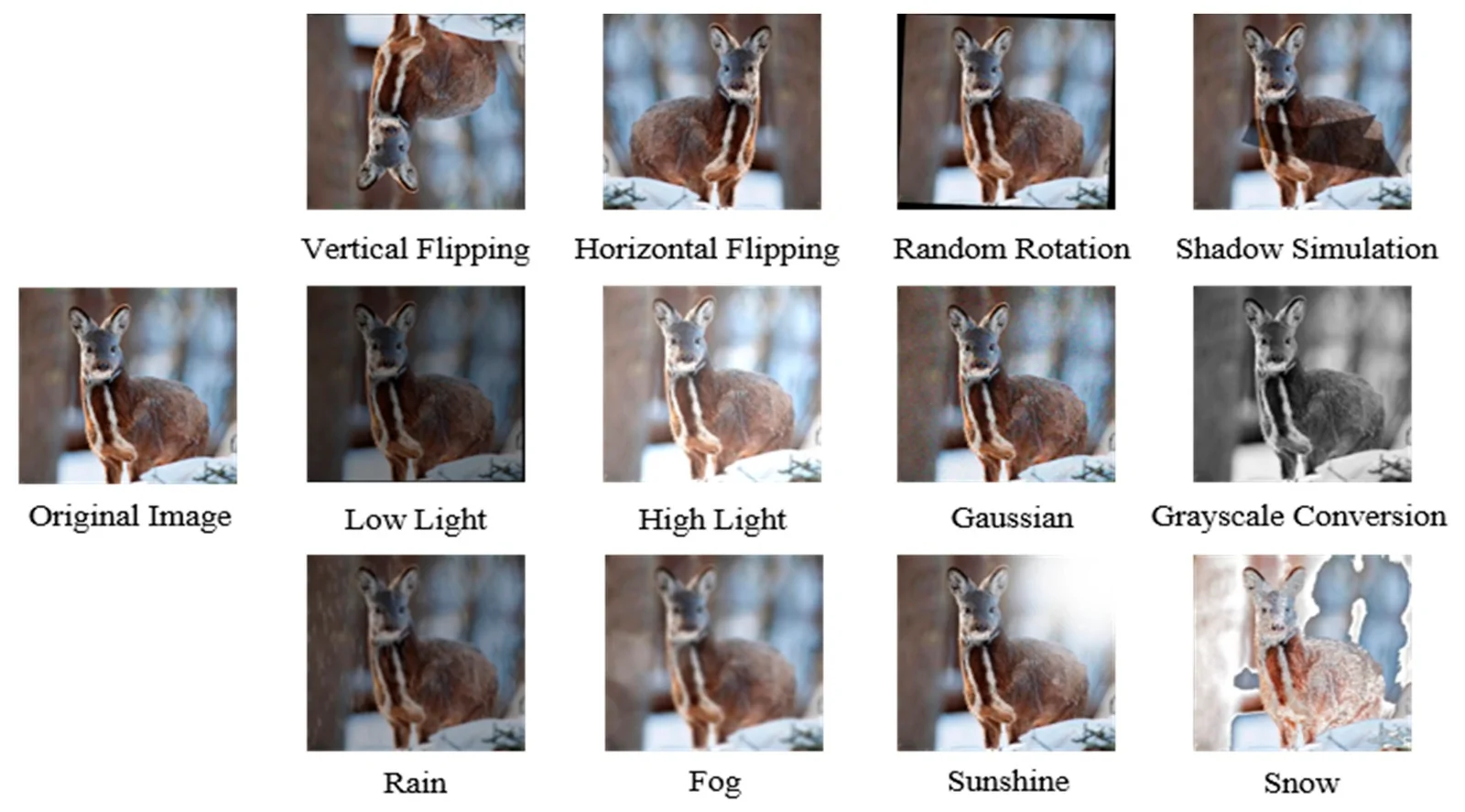
Examples of data augmentation applied to the wildlife dataset.

To further improve model robustness and alleviate overfitting caused by limited wildlife samples, several data augmentation strategies were employed during training. In addition to common geometric and photometric transformations, such as random flipping, rotation, brightness adjustment, and blur simulation, weather- and illumination-related augmentations were introduced to mimic the variable environmental conditions commonly encountered in field monitoring images. These transformations were designed to simulate changes in animal posture, imaging angle, light intensity, motion blur, shadow interference, and adverse weather conditions, thereby improving the adaptability of the model to complex ecological scenarios (Figure 3). In addition, RT-DETR-r18 model was trained with and without data augmentation, and the two models were evaluated on the same test set. The model trained with data augmentation showed better overall performance, particularly in precision and mAP50, indicating that the data augmentation strategy was beneficial for model training (Table S3).

Finally, the dataset was randomly divided into training, validation, and test subsets with an approximate ratio of 7:1:2 for model development and evaluation.

### 2.2. Overall Architecture of DSCF-DET

**Figure 4:**
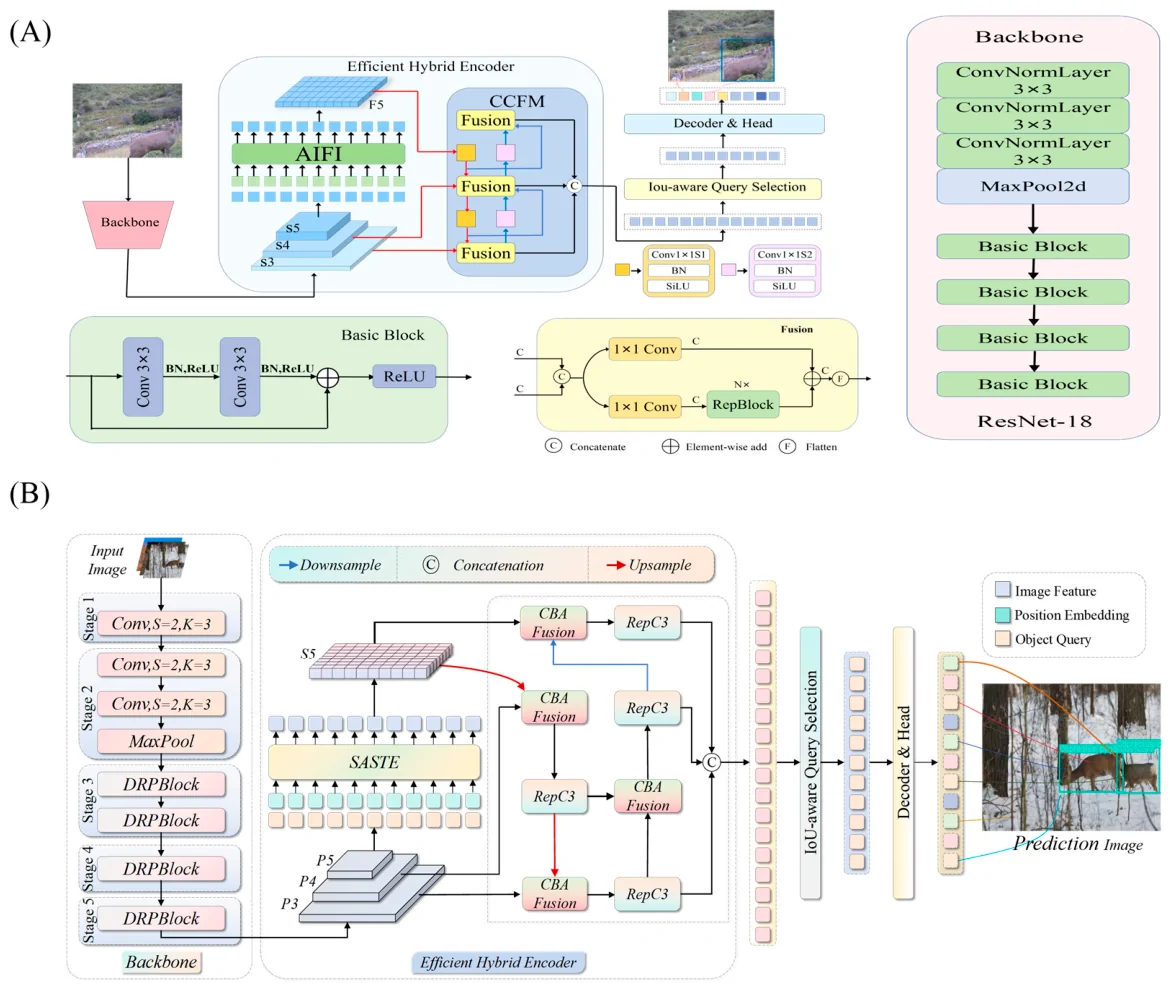
Overall architectures of the baseline RT-DETR and the proposed DSCF-DET. (**A**) The baseline RT-DETR architecture. (**B**) The proposed DSCF-DET architecture, incorporating DRPBlock, SASTE, and CBAFusion into the backbone, encoder, and cross-scale feature-fusion stages, respectively.

The baseline RT-DETR used in this study consists of a ResNet series backbone for multi-scale feature extraction, an attention-based encoding component for high-level semantic modeling, a cross-scale feature fusion pathway, and a transformer decoder for object classification and localization. Building on this architecture, DSCF-DET introduces targeted modifications to the backbone, encoder, and feature fusion pathway through DRPBlock, SASTE, and CBAFusion, respectively. The overall architectures of RT-DETR and DSCF-DET are presented separately (Figure 4A,B). The input image first passes through shallow convolution and normalization layers for initial feature extraction. It is then processed by a hierarchical backbone composed of Dilated Receptive-Field Perception Blocks (DRPBlocks). In each residual stage, local and surrounding-context branches model spatial dependencies with depthwise convolutions parameterized by a shared dilation setting. The fused features are recalibrated by the Cascaded Channel-Spatial Recalibration Unit (CCSRUnit), generating multi-scale P3, P4, and P5 feature maps. The P5 feature map is projected by a pointwise convolution and passed to the Sparse-Aware Spatial Transformer Encoder (SASTE) for global semantic encoding. SASTE replaces standard multi-head self-attention with Window-Guided Sparse Self-Attention (WGSSA), replaces the standard feed-forward network with a Spatial-Prior Gated Feed-forward Network (SPGFN), and adds Multi-Kernel Residual Adapter (MKRA) modules for local multi-scale compensation. The encoded features are propagated bidirectionally through the neck. At each cross-level fusion node, the original concatenation operation is replaced by Cross-Level Bilinear Attention Fusion (CBAFusion), which combines bilinear cross-scale coupling with Prompt-Guided Patch Attention (PGPA). The resulting P3, P4, and P5 features are finally fed into the RT-DETR decoder for object classification and localization.

#### 2.2.1. Dilated Receptive-Field Perception Block

**Figure 5:**
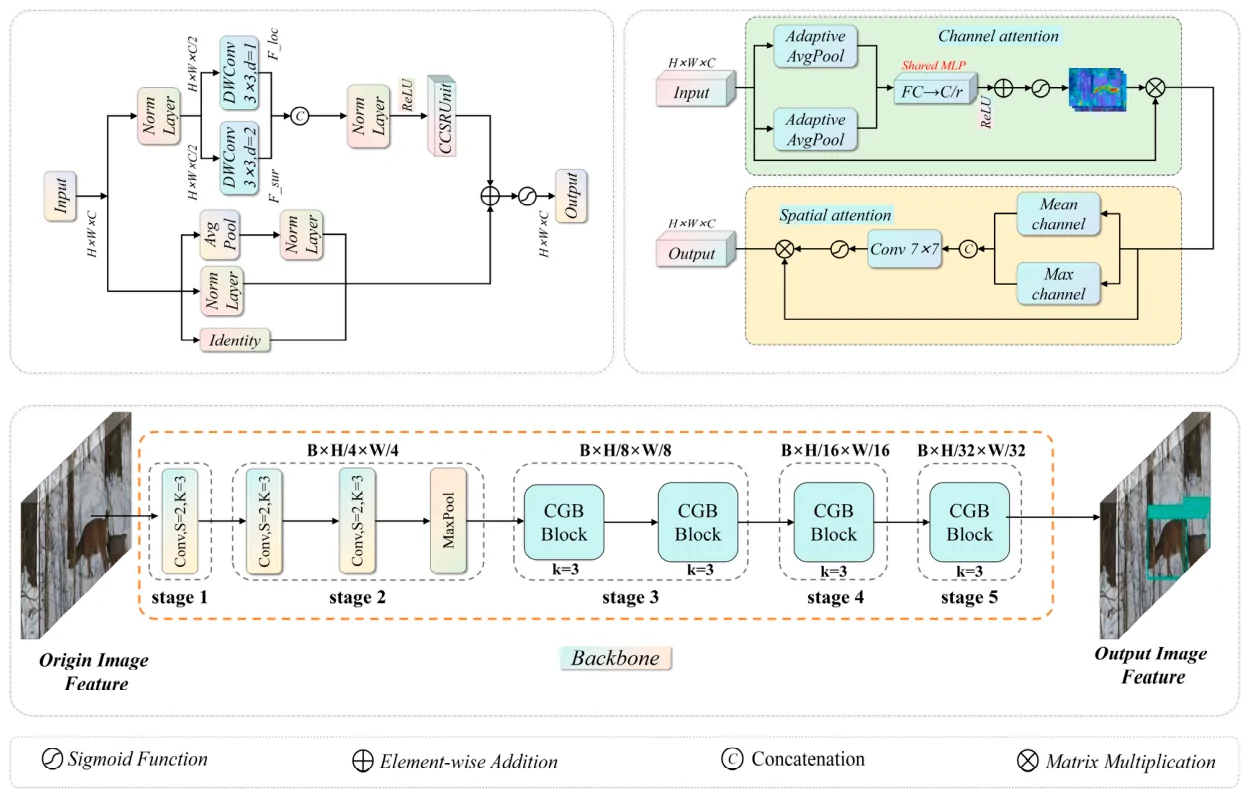
Structure of DRPBlock and CCSRUnit, including the local and surrounding-context convolution branches and the cascaded channel-spatial recalibration unit.

This subsection introduces the proposed DRPBlock, which is used to replace the original ResNet backbone of RT-DETR. In the conventional ResNet backbone, feature extraction mainly relies on stacked convolutions with fixed kernel sizes and fixed dilation rates. This design provides a limited receptive field within each unit and is not well suited to jointly modeling fine local textures and surrounding contextual information. The original residual structure also lacks explicit channel- and spatial-wise guidance, which may cause feature confusion among artiodactyl species with similar body contours and coat textures. To address these limitations, a lightweight backbone based on DRPBlock was designed. The block introduces parallel local and surrounding-context branches into each residual unit and applies dual attention recalibration to the fused features. This design strengthens responses in discriminative regions while maintaining a compact backbone (Figure 5).

DRPBlock preserves the overall topology of the original residual structure and reconstructs the main branch as a feature-extraction unit for joint local and surrounding-context perception. Given an intermediate feature map $F_{1}\in R^{H\times W\times C}$ after the initial convolutional transformation, the local and surrounding-context branches can be described as depthwise separable convolution operators parameterized by the dilation rate r. The response at spatial position p is expressed as follows:

(1) $$O_{r}\left(p,c\right)=\sum_{\Delta \in K}w_{r}\left(\Delta ,c\right)\;F_{1}\left(p+r\Delta ,\;c\right),r\in \{1,2\}$$

where K denotes the offset set of the 3 × 3 convolution kernel, and $w_{r}\left(\Delta ,c\right)$ is the learnable kernel weight of the c-th channel under the corresponding dilation rate. The setting r=1 corresponds to the local perception branch $F_{loc}$, whereas r=2 corresponds to the surrounding-context branch $F_{sur}$. These two branches cover adjacent and extended neighborhoods with the same parameter scale. The outputs $O_{1}$ and $O_{2}$ are concatenated along the channel dimension and then processed by one-dimensional convolution, batch normalization, and activation to obtain the joint feature $F_{joi}$. The CCSRUnit operator $\Phi \left(\cdot \right)$ then recalibrates the feature in channel and spatial dimensions, and the residual mapping of the module is defined as follows:

(2) $$Y\left(p,c\right)=\delta \left(\Phi \left(F_{joi}\right)\left(p,c\right)+S\left(X\right)\left(p,c\right)\right)$$

where $S\left(\cdot \right)$ denotes the identity or dimensionality-reduction shortcut, and $\delta \left(\cdot \right)$ denotes the final activation function. By embedding the dilation rate into a unified convolutional operator, the module decouples local and surrounding-context modeling with limited additional computation. The recalibration operator Φ further improves the discriminative representation of the features. DRPBlock differs from many standard dilated-convolution or dilated residual designs in how local and contextual information is organized. In many dilation-based modules, the dilation rate mainly changes the sampling interval within a single feature path. DRPBlock instead separates local texture modeling and surrounding-context modeling into two parallel branches, while keeping the dilation-related convolutional formulation unified. After branch fusion, CCSRUnit recalibrates the features using both global average and maximum statistics along channel and spatial dimensions. Rather than serving as an independent plug-in after feature extraction, as in generic channel-spatial attention modules such as CBAM or coordinate attention [26], this recalibration is coupled with the local-context decomposition of DRPBlock. This coupling helps strengthen target-related responses and mitigate background-dominated activations in camouflaged wildlife scenes.

CCSRUnit sequentially performs channel and spatial recalibration on the joint feature $F_{joi}\in R^{H\times W\times C}$. The channel attention weights are determined by the first-order global statistic and the maximum statistic of the feature. Let $z_{c}^{avg}=\frac{1}{HW}\sum_{i=1}^{H}\sum_{j=1}^{W}F_{joi}\left(i,j,c\right)$ and $z_{c}^{max}={max}_{\left(i,j\right)}F_{joi}\left(i,j,c\right)$ denote the global average and global maximum of the c-th channel, respectively. The channel attention vector is defined as follows:

(3) $$M_{c}=\sigma \left(W_{2}\;\delta \left(W_{1}z^{avg}\right)+W_{2}\;\delta \left(W_{1}z^{max}\right)\right)$$

where $W_{1},W_{2}$ are learnable mapping matrices for channel reduction and restoration, and $\delta \left(\cdot \right)$ and $\sigma \left(\cdot \right)$ denote the ReLU and sigmoid functions, respectively. After weighting by $M_{c}$, the channel-refined feature is obtained as $F_{c}=F_{joi}⊙M_{c}$. Spatial attention is then derived from statistical aggregation along the channel dimension followed by local-neighborhood convolution. Let $s^{avg}\left(i,j\right)=\frac{1}{C}\sum_{c=1}^{C}F_{c}\left(i,j,c\right)$ and $s^{max}\left(i,j\right)={max}_{c}F_{c}\left(i,j,c\right)$ denote the average and maximum spatial descriptors, respectively. The spatial attention map is expressed as follows:

(4) $$M_{s}\left(i,j\right)=\sigma \left(\sum_{\left(u,v\right)\in N_{7\times 7}}k\left(u,v\right)\cdot [s^{avg};s^{max}]\left(i+u,j+v\right)\right)$$

where $N_{7\times 7}$ is the offset set of the 7 × 7 neighborhood, and $k\left(\cdot ,\cdot \right)$ denotes the corresponding convolution kernel weight. Channel attention and spatial attention are applied in series to the joint feature, and the final output is formulated as follows:

(5) $$F_{out}\left(i,j,c\right)=F_{joi}\left(i,j,c\right)\cdot M_{c}\left(c\right)\cdot M_{s}\left(i,j\right)$$

This cascaded structure first selects target-relevant semantic channels and then focuses on discriminative spatial regions, thereby suppressing redundant background information and enhancing key features. DRPBlock uses dilation-parameterized dual-branch convolutions to decouple local details from surrounding contextual information within each residual unit. CCSRUnit further recalibrates the fused features across channel and spatial dimensions. Together, these components improve multi-scale contextual perception and fine-grained feature representation in the backbone, providing more discriminative features for the subsequent detection head.

#### 2.2.2. Sparse-Aware Spatial Transformer Encoder

**Figure 6:**
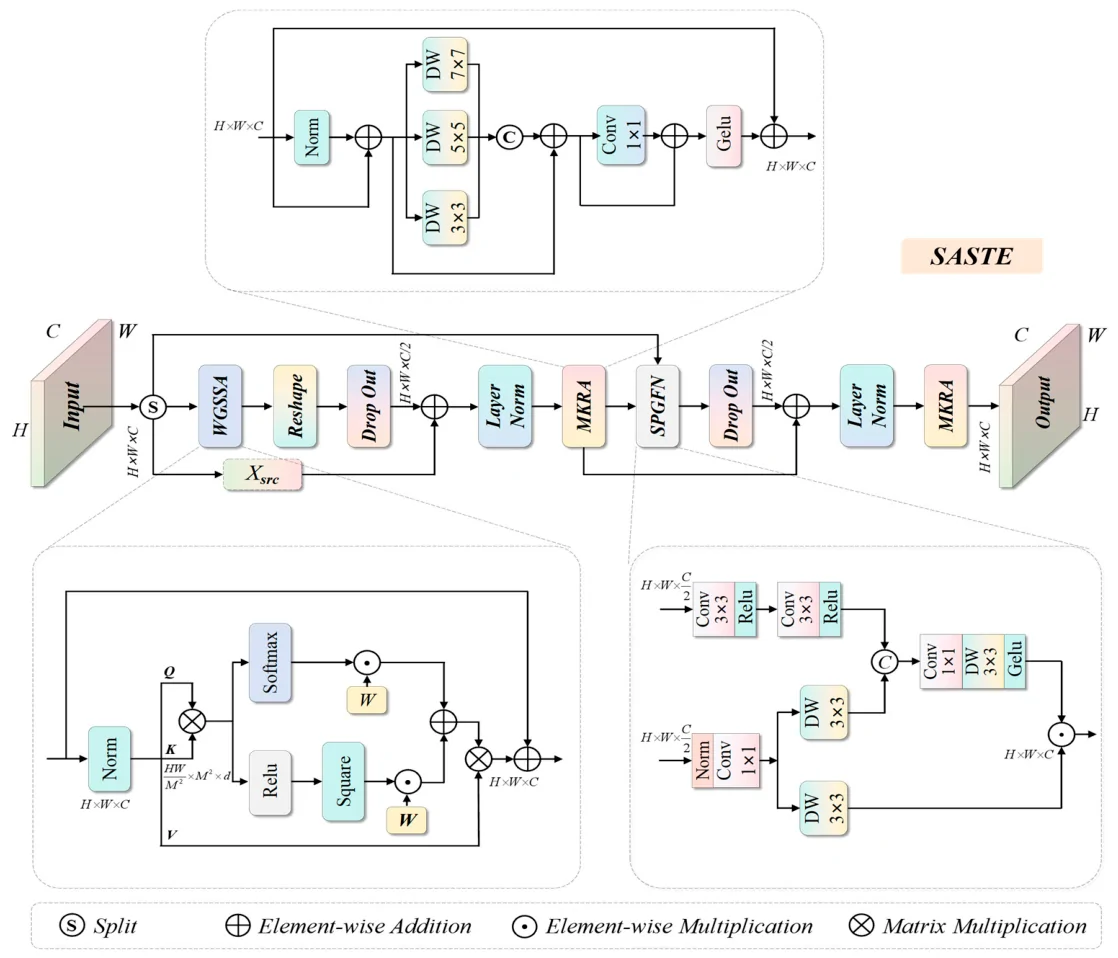
Structure of SASTE, including WGSSA, SPGFN, and MKRA.

SASTE was designed to replace the dense transformer encoder layer of the baseline RT-DETR. The standard TransformerEncoderLayer used in the RT-DETR encoder consists of dense multi-head self-attention and a fully connected feed-forward network. Dense self-attention computes pairwise similarities over all spatial tokens, which gives quadratic complexity with respect to token number and may allocate attention to irrelevant background regions. The standard feed-forward network mainly performs channel-wise transformations and does not explicitly model spatial structure. To improve the representation of local texture and contour differences among visually similar artiodactyls, SASTE was introduced. It uses WGSSA for sparse window attention, SPGFN for spatially guided feed-forward transformation, and MKRA for lightweight multi-scale refinement (Figure 6).

SASTE adopts a post-normalization residual structure with two sublayers. The attention and feed-forward sublayers are replaced by the WGSSA operator $A_{sparse}\left(\cdot \right)$ and the SPGFN operator $F_{SEFN}\left(\cdot ,\cdot \right)$, respectively. An MKRA operator $M\left(\cdot \right)$ is introduced after the normalization step in each sublayer. For the input feature $X\in R^{B\times C\times H\times W}$, the attention sublayer is formulated as follows:

(6) $$X_{1}=M_{1}\left({LN}_{1}\left(X+A_{sparse}\left(X\right)\right)\right)$$

where ${LN}_{1}$ denotes layer normalization along the channel dimension. The operator $A_{sparse}\left(\cdot \right)$ flattens the input into a token sequence, performs adaptive sparse window attention, and restores the original spatial shape. The operator $M_{1}\left(\cdot \right)$ is the first MKRA module, which applies multi-scale convolutional correction to the attention-refined feature. In the feed-forward sublayer, SPGFN receives the attention-refined feature $X_{1}$ and the original input X as the spatial guidance branch. The overall output is written as follows:

(7) $$Y=M_{2}\left({LN}_{2}\left(X_{1}+F_{SEFN}\left(X_{1},\;X\right)\right)\right)$$

where $F_{SEFN}\left(X_{1},X\right)$ uses $X_{1}$ as the main feature and X as the auxiliary spatial prior for gated fusion, and $M_{2}\left(\cdot \right)$ denotes the second MKRA operator. By combining sparse attention, spatially guided feed-forward transformation, and lightweight adapters in a residual cascade, the encoder maintains the original Transformer residual topology while modeling sparse global dependencies and compensating local spatial details.

WGSSA performs adaptive sparse self-attention in a window-based manner. For an input feature $X\in R^{B\times C\times H\times W}$, the feature is first flattened into a token sequence, normalized, and partitioned into windows of size P×P. For each window $X_{w}\in R^{P^{2}\times C}$, query, key, and value projections are computed, and an attention matrix with relative positional bias and a shifted-window mask is constructed. Adaptive sparse gating is then used to retain informative attention weights, as follows:

(8) $$A_{w}=softmax\left(\frac{Q_{w}K_{w}^{⊤}}{\sqrt{d}}+B+M_{shift}\right)⊙S\left(Q_{w}K_{w}^{⊤}\right),O_{w}=A_{w}V_{w}$$

where $Q_{w},K_{w},V_{w}$ denote the query, key, and value matrices within a window, and d is the feature dimension of each attention head. The terms B and $M_{shift}$ denote the relative positional bias and shifted-window attention mask, respectively. The function $S\left(\cdot \right)$ is generated from attention similarity to suppress low-correlation responses and retain more discriminative attention connections. The window-attention output is restored to the original token sequence, connected to the input through a residual path, and then passed through layer normalization and the feed-forward module:

(9) $$X^{\prime }=X+W_{sparse}\left(LN\left(X\right)\right),X^{\prime \prime }=X^{\prime }+FRFN\left(LN\left(X^{\prime }\right)\right)$$

where $W_{sparse}\left(\cdot \right)$ denotes the complete operator consisting of window partitioning, sparse attention computation, and window reconstruction, and $FRFN\left(\cdot \right)$ denotes the feed-forward network. The combination of window partitioning and sparsification makes attention focus on highly correlated local regions while reducing computational complexity.

SPGFN introduces an independent spatial branch into the standard gated feed-forward network to enhance spatial-context perception during channel transformation. Given the main input feature X and the spatial guidance feature $X_{s}$, the main branch first uses pointwise convolution to project the feature into twice the hidden dimension and then divides it into two parallel subfeatures $X_{1},X_{2}$. In parallel, the spatial branch downsamples $X_{s}$, applies multiple convolution-normalization transformations, and upsamples it back to the original resolution. This process is expressed as follows:

(10) $$Y_{s}=Up\left(C_{\theta }\left({AvgPool}_{2\times 2}\left(X_{s}\right)\right)\right)$$

where $C_{\theta }\left(\cdot \right)$ denotes a spatial feature-refinement operator composed of convolution, layer normalization, and nonlinear activation. The operator $Up\left(\cdot \right)$ denotes upsampling, and $Y_{s}$ is used as the guidance feature carrying low-frequency spatial priors. Subsequently, $X_{1}$ and $Y_{s}$ are concatenated along the channel dimension and processed by fusion convolution and depthwise convolution. The result is multiplied with $X_{2}$ through a gating operation to obtain the final output:

(11) $$X_{out}=W_{out}\left(GELU\left(D\left(F\left(\left[X_{1};Y_{s}\right]\right)\right)\right)⊙X_{2}\right)$$

where $\left[X_{1};Y_{s}\right]$ denotes concatenation along the channel dimension, $F\left(\cdot \right)$ is the fusion convolution, $D\left(\cdot \right)$ is the depthwise convolution after fusion, $W_{out}\left(\cdot \right)$ is the output projection convolution, and ⊙ denotes element-wise multiplication. This design incorporates low-frequency spatial context into channel gating and improves the spatial consistency of the feature representation.

MKRA is a lightweight adapter that recombines normalized and original features through learnable scaling coefficients. It then performs multi-scale convolutional aggregation in a low-dimensional bottleneck space before projecting the feature back to the original channel dimension. For an input feature X, learnable parameters γ and $\gamma_{x}$ are used to combine the layer-normalized feature and the original feature, producing the modulated representation:

(12) $$\tilde{X}=\gamma ⊙LN\left(X\right)+\gamma_{x}⊙X$$

where $\gamma ,\gamma_{x}\in R^{C\times 1\times 1}$ are learnable scaling parameters broadcast along the channel dimension, and $LN\left(\cdot \right)$ denotes layer normalization. The modulated feature is projected to a low-dimensional space as $Z=W_{1}\tilde{X}$. In this space, depthwise convolutions with different kernel sizes are applied in parallel to aggregate local multi-scale context. Two residual connections and pointwise projection are then used for refinement, and the final output is expressed as follows:

(13) $$X_{out}=X+W_{2}\left(Drop\left(GELU\left(Z^{\prime }+P_{1\times 1}\left(Z^{\prime }\right)\right)\right)\right),\;$$

(14) $$Z^{\prime }=Z+\frac{1}{3}\sum_{k\in \{3,5,7\}}{DWConv}_{k\times k}\left(Z\right)$$

where ${DWConv}_{k\times k}$ denotes a depthwise separable convolution with kernel size k, $P_{1\times 1}\left(\cdot \right)$ is the pointwise re-projection in the low-dimensional space, and $W_{1},W_{2}$ are the dimensionality-reduction and dimensionality-restoration projection matrices, respectively. By performing multi-scale convolutional aggregation and residual refinement in a bottleneck space, MKRA provides local multi-scale compensation with a small parameter overhead and complements WGSSA and SPGFN.

In summary, SASTE replaces dense global attention with WGSSA to reduce background interference and focus on informative local regions. SPGFN introduces low-frequency spatial context into the feed-forward transformation, whereas MKRA provides lightweight local multi-scale compensation. These components preserve the residual topology of the original Transformer while improving the representation of fine-grained local patterns. Compared with standard window self-attention schemes such as Swin Transformer [27], where attention is mainly computed within predefined local windows, WGSSA in SASTE introduces adaptive sparse gating to weaken low-relevance background token interactions during attention calculation. Meanwhile, the spatial-prior guided SPGFN extends the conventional position-wise feed-forward transformation by incorporating spatial-prior modulation, and the lightweight MKRA provides additional multi-kernel local compensation for fine-grained pattern representation. These designs make SASTE more selective than a direct window-attention encoder while retaining the efficiency advantage of local-window modeling.

#### 2.2.3. Cross-Level Bilinear Attention Fusion

**Figure 7:**
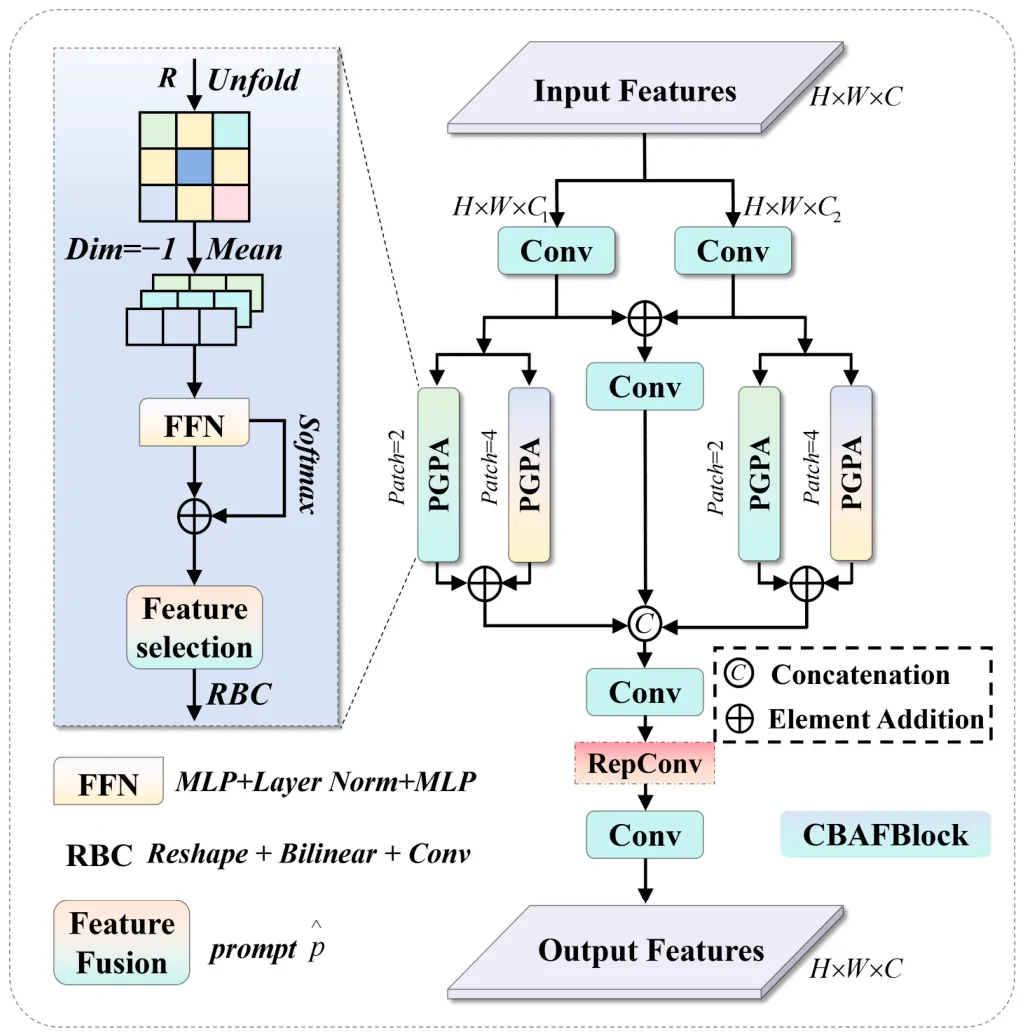
Structure of CBAFusion, including the bilinear coupling path and PGPA.

To overcome the limitations of the simple fusion operation in the original RT-DETR neck, this study introduces CBAFusion as a cross-scale fusion node in DSCF-DET. In the neck of RT-DETR, common feature-fusion strategies such as element-wise addition or channel concatenation treat multi-scale features with fixed and uniform fusion patterns. Such operations are limited in distinguishing local details from global semantics across feature levels and may weaken fine-grained cues during fusion. To address this problem, CBAFusion was designed for cross-level fusion nodes. It constructs local-global attention representations for each input branch and uses a bilinear coupling path to model implicit interactions between scales. The fused features are then processed by a unified convolutional re-parameterization structure (Figure 7).

CBAFusion consists of two parallel paths, namely cross-scale projection coupling and hierarchical attention representation. For input features $x_{1}\in R^{H\times W\times C_{1}}$ and $x_{2}\in R^{H\times W\times C_{2}}$ from different levels, pointwise projection $\Psi_{i}\left(\cdot \right)$ first maps them to a unified hidden dimension $h_{idc}$. A grouped convolution is then used to construct the bilinear coupling representation:

(15) $$B\left(x_{1},x_{2}\right)=W_{3\times 3,g=4}\left(\Psi_{1}\left(x_{1}\right)⊕\Psi_{2}\left(x_{2}\right)\right)$$

where $\Psi_{i}\left(x_{i}\right)=W_{1\times 1}^{\left(i\right)}\left(x_{i}\right)$ denotes the pointwise projection of the i-th input branch, ⊕ denotes element-wise addition, and $W_{3\times 3,g=4}$ is a 3 × 3 convolution with four groups. The coupling term $B\left(x_{1},x_{2}\right)$ implicitly models the joint distribution of the two scale features in the hidden space through additive interaction. In parallel, $\Psi_{1}\left(x_{1}\right)$ and $\Psi_{2}\left(x_{2}\right)$ are processed by the PGPA operator $A\left(\cdot \right)$ to obtain the hierarchical attention representations $A\left(\Psi_{1}\left(x_{1}\right)\right)$ and $A\left(\Psi_{2}\left(x_{2}\right)\right)$, respectively. The three feature streams are concatenated along the channel dimension and then processed by channel compression, re-parameterized convolution, and final pointwise convolution to produce the output:

(16) $$Y=f_{final}\left(f_{rep}\left(f_{sq}\left[A\left(\Psi_{1}\left(x_{1}\right)\right);|A\left(\Psi_{2}\left(x_{2}\right)\right);|B\left(x_{1},x_{2}\right)\right]\right)\right)$$

where $f_{sq}$ denotes the channel-compression convolution, $f_{rep}$ denotes the grouped re-parameterized convolution, $f_{final}$ denotes the output projection convolution, and $\left[\cdot ;\cdot ;\cdot \right]$ denotes concatenation along the channel dimension. This structure refines each input branch with attention while preserving the overall cross-scale relationship through the bilinear coupling term $B\left(x_{1},x_{2}\right)$.

PGPA models local spatial statistics and global semantic priors through a patch-based process. For an input feature $x\in R^{H\times W\times C}$, the feature is first divided into non-overlapping windows of size P×P. The channel-wise mean of each window is used to obtain a local descriptor ${\bar{x}}_{n}\in R^{P^{2}}$. This descriptor is mapped to the output dimension by a two-layer perceptron and layer normalization and is then recalibrated by self-attention:

(17) $$z_{n}={MLP}_{2}\left(LN\left({MLP}_{1}\left({\bar{x}}_{n}\right)\right)\right),o_{n}=z_{n}⊙softmax\left(z_{n}\right)$$

where n is the window index, and ${\bar{x}}_{n}$ denotes the mean vector of features in the n-th window. The operators ${MLP}_{1}$ and ${MLP}_{2}$ perform dimensionality reduction and restoration, respectively. The operator $softmax\left(\cdot \right)$ computes the attention distribution along the output-channel dimension, and ⊙ denotes element-wise multiplication. This step adaptively assigns importance to each window representation. A task-related learnable prompt vector $p\in R^{output\_dim}$ is then introduced. The cosine similarity between the window feature and the prompt vector generates a gating coefficient, and the learnable linear transformation matrix T maps the gated feature in a high-dimensional space:

(18) $${\hat{o}}_{n}=\left(o_{n}⊙clamp\left(cos\left(o_{n},\;p\right),\;0,\;1\right)\right)\;T$$

where $cos\left(o_{n},p\right)=\frac{o_{n}\cdot p}{∥o_{n}∥\;∥p∥}$ denotes the cosine similarity between the window feature and the prompt vector. The function $clamp\left(\cdot ,0,1\right)$ constrains this similarity to the interval from 0 to 1 as a soft-gating coefficient. The matrix $T\in R^{output\_dim\times output\_dim}$ is a learnable topological transformation matrix for remapping the gated feature in channel space. The transformed window-level feature is restored to the original spatial resolution by bilinear interpolation and then passed through a pointwise convolution. This process introduces discriminative guidance consistent with the global semantic prior while preserving spatial detail.

In summary, CBAFusion combines a bilinear coupling path with hierarchical PGPA to preserve fine spatial details and introduce task-related global semantic guidance during multi-scale feature fusion. The bilinear term improves cross-scale consistency, whereas PGPA uses window-level statistics, self-attention recalibration, and prompt-guided gating to focus the fused features on discriminative regions. This design provides the detection head with more informative multi-scale features for distinguishing visually similar artiodactyl species. Distinct from element-wise addition in FPN or concatenation-based aggregation in PANet [28], CBAFusion constructs bilinear cross-scale coupling paths to model feature interactions between different scale layers. With prompt-guided patch attention (PGPA), the fusion process further emphasizes informative patch-level responses during cross-scale feature integration. Compared with weighted fusion strategies such as BiFPN and adaptive spatial fusion methods such as ASFF [29,30], CBAFusion focuses on coupling cross-level feature interaction with attention-guided patch selection, thereby providing more discriminative fused features for visually similar artiodactyl species.

### 2.3. Training Settings and Evaluation Metrics

All experiments were conducted in a unified software and hardware environment (Table 2). The input images were resized to 640 × 640 pixels. The batch size was set to 4, the initial learning rate was 0.01, and all models were trained for 150 epochs. The same dataset partition, data augmentation strategy, and evaluation procedure were used throughout the comparative and ablation experiments.

For the detector comparison, the YOLO baselines were trained using the official implementations released by their respective developers. The RT-DETR baselines and the proposed DSCF-DET were implemented within the Ultralytics framework. The Faster R-CNN baseline adopted the VGG16-based configuration. The native architectural components and loss formulations of the baseline detectors were retained, and all compared detectors were evaluated under the common experimental protocol described above. The backbone comparison was conducted within the same RT-DETR framework. Only the feature-extraction network was replaced, whereas the encoder, feature-fusion neck, decoder, dataset partition, training procedure, data augmentation strategy, and evaluation protocol remained unchanged.

To comprehensively evaluate detection performance and computational efficiency, precision, recall, F1-score, mean average precision at an IoU threshold of 0.5 (mAP50), parameter count (Params) and GFLOPs were adopted as evaluation metrics.

Precision and recall were defined as:

(19) $$Precision=\frac{TP}{TP+FP}$$

(20) $$Recall=\frac{TP}{TP+FN}$$

where TP, FP, and FN denote true positives, false positives, and false negatives, respectively.

The F1-score is calculated as:

(21) $$F1=\frac{2\times Precision\times Recall}{Precision+Recall}$$

The mean average precision at an IoU threshold of 0.5 is defined as:

(22) $$mAP50=\frac{1}{N}\sum_{i=1}^{N}{AP}_{i}$$

where $AP_{i}$ denotes the average precision of the i-th category and N represents the total number of categories.

In addition to detection accuracy, model complexity and inference efficiency were also evaluated. Params and GFLOPs were used to measure structural complexity and computational cost, respectively.

## 3. Results

### 3.1. Comparative Experiments

#### 3.1.1. Detector Comparison

**Figure 8:**
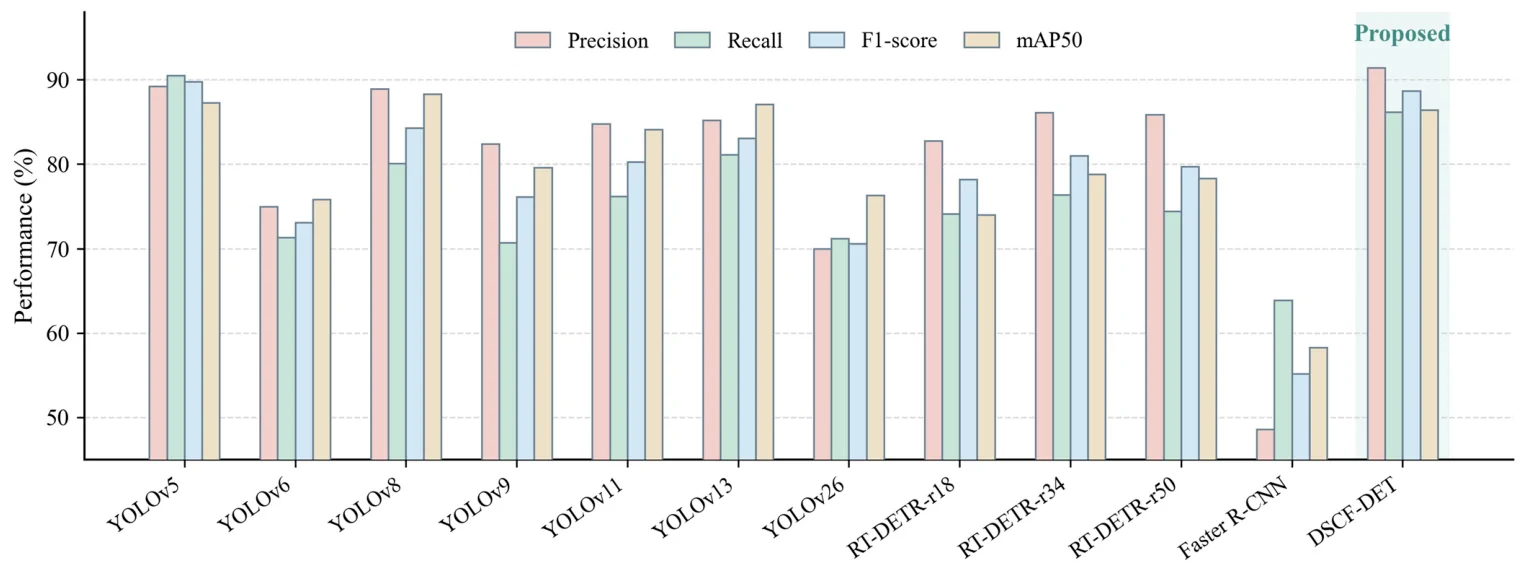
Visual comparison of detection accuracy metrics across different object detection models.

Under the training settings described in Section 2.3, DSCF-DET was evaluated on the fine-grained wildlife dataset against representative YOLO-based, RT-DETR-based, and two-stage detectors (Table 3 and Figure 8). It achieved a precision of 91.4%, a recall of 86.2%, an F1-score of 88.7%, and an mAP50 of 86.4%. The highest values were not concentrated in a single model: YOLOv5 showed the highest recall (90.5%) and F1-score (89.8%), whereas DSCF-DET showed the highest precision. Relative to YOLOv8 and YOLOv13, DSCF-DET increased the F1-score by 4.4 and 5.6 percentage points, respectively. The corresponding model size and computational cost were 23.7 M parameters and 64.4 GFLOPs.

The advantage of DSCF-DET was more evident when compared with the RT-DETR baseline. Relative to RT-DETR-r18, DSCF-DET improved precision, recall, F1-score, and mAP50 by 8.6, 12.1, 10.5, and 12.4 percentage points, respectively, with only moderate increases in parameters and GFLOPs. It also substantially outperformed RT-DETR-r34 and RT-DETR-r50 in all accuracy metrics while using fewer parameters and less computation. Faster R-CNN showed the largest model size and computational cost but produced the lowest detection performance, indicating that simply increasing model complexity did not lead to better results on this dataset. Overall, DSCF-DET provided a favorable balance between detection accuracy and computational demand for fine-grained wildlife detection.

#### 3.1.2. Backbone Comparison

To evaluate the effectiveness of DRPBlock as the feature extraction backbone, several representative backbone networks were incorporated into the same RT-DETR detection framework and evaluated under identical experimental settings.

DRPBlock achieved the best overall detection performance among all compared backbones, with 89.6% precision, 82.9% recall, 86.1% F1-score, and 83.2% mAP50. Relative to the original ResNet-18 backbone, DRPBlock improved precision, recall, F1-score, and mAP50 by 6.8, 8.8, 7.9, and 9.2 percentage points, respectively. Meanwhile, the number of parameters decreased from 19.9 M to 17.5 M, and the computational cost decreased from 57.0 G to 50.3 G (Table 4).

### 3.2. Ablation Experiments

#### 3.2.1. Overall Contribution of Each Module

**Figure 9:**
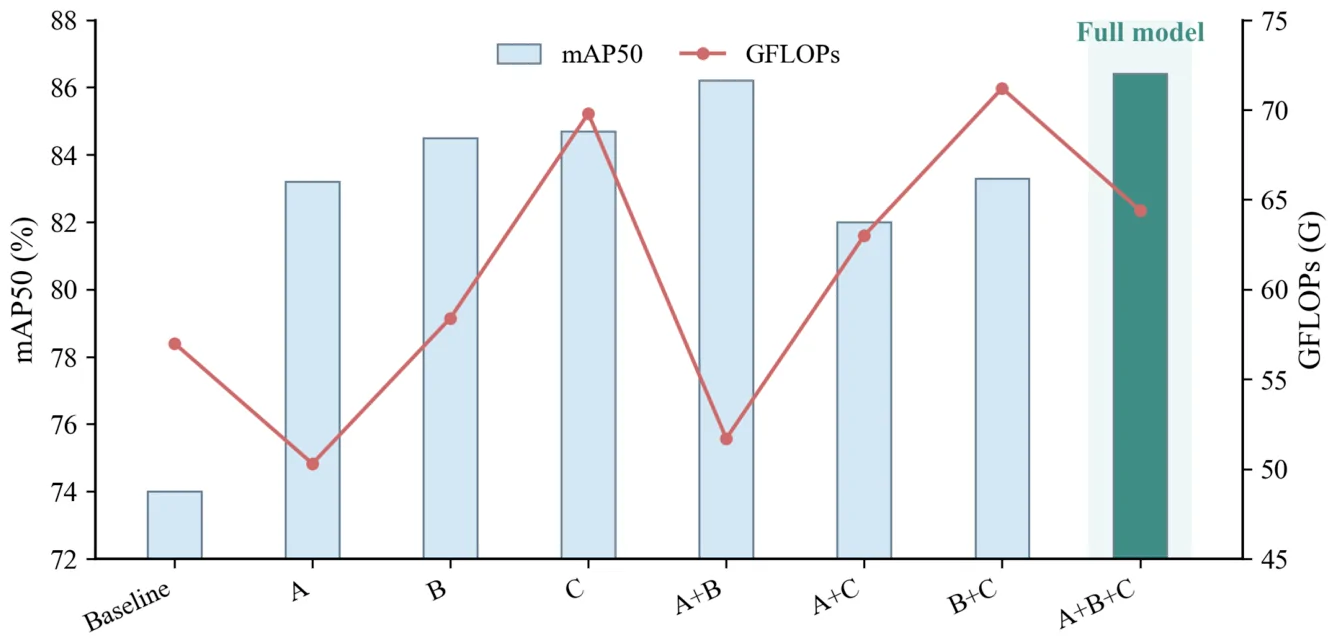
Ablation analysis of the proposed modules in terms of mAP50 and GFLOPs.

The complete ablation results showed that A, B, and C, denoting DRPBlock, SASTE, and CBAFusion, respectively, had different effects on detection accuracy and computational cost (Table 5 and Figure 9). Adding DRPBlock alone increased mAP50 from 74.0% to 83.2% while reducing GFLOPs from 57.0 G to 50.3 G, indicating that this module improved feature representation without increasing computational cost. SASTE and CBAFusion also improved mAP50 when introduced independently, reaching 84.5% and 84.7%, respectively, although CBAFusion required a larger increase in GFLOPs.

The combination of DRPBlock and SASTE provided the most efficient two-module configuration, achieving an mAP50 of 86.2% with only 51.7 GFLOPs. In contrast, adding CBAFusion without the full module set led to higher computational costs but less consistent accuracy gains, particularly for the B + C setting. After integrating all three modules, the full DSCF-DET model achieved the highest mAP50 of 86.4% and maintained a moderate computational cost of 64.4 GFLOPs. These results indicate that DRPBlock and SASTE contribute the main accuracy-efficiency gains, while CBAFusion further improves performance when combined with the complete architecture.

#### 3.2.2. Ablation of DRPBlock

The baseline model without $F_{loc}$, $F_{sur}$, or CCSRUnit achieved a precision of 82.8%, a recall of 74.1%, an F1-score of 78.2%, and an mAP50 of 74.0%. After introducing $F_{loc}$, all accuracy metrics improved, with precision, recall, F1-score, and mAP50 increasing to 85.7%, 78.6%, 82.0%, and 80.7%, respectively. This variant reduced the parameter count from 19.9 M to 13.6 M and decreased GFLOPs from 57.0 G to 42.0 G, suggesting that the local feature branch enhanced feature extraction efficiency without increasing computational complexity (Table 6).

Adding $F_{sur}$ further increased the precision, recall, F1-score, and mAP50 to 86.6%, 80.0%, 83.2%, and 81.7%, respectively. This improvement indicates that surrounding-context information complements local texture cues and helps the model distinguish visually similar animal categories. The full DRPBlock, which integrates $F_{loc}$, $F_{sur}$, and CCSRUnit, achieved the best overall performance, with a precision of 89.6%, a recall of 82.9%, an F1-score of 86.1%, and an mAP50 of 83.2%. Compared with the baseline, the complete module improved precision by 6.8 percentage points, recall by 8.8 percentage points, F1-score by 7.9 percentage points, and mAP50 by 9.2 percentage points, while maintaining fewer parameters and lower GFLOPs than the baseline. These results demonstrate that dual-branch receptive-field modeling and cascaded channel-spatial recalibration jointly enhance fine-grained feature representation.

#### 3.2.3. Ablation of SASTE

To examine the incremental effects of WGSSA, SPGFN, and MKRA, all SASTE variants were evaluated using the same baseline encoder (Table 7). Under the current experimental setting, replacing MHSA with WGSSA was associated with a slight increase in precision from 82.8% to 83.9% and mAP50 from 74.0% to 78.2%, whereas recall remained nearly unchanged.

When SPGFN was further introduced, precision, recall, F1-score, and mAP50 increased to 86.7%, 78.7%, 82.5%, and 81.7%, respectively. Compared with the WGSSA-only variant, SPGFN increased recall by 4.5 percentage points and mAP50 by 3.5 percentage points, suggesting that the spatial-prior guided feed-forward design strengthened spatial consistency and improved the detection of difficult targets. The complete SASTE, which integrates WGSSA, SPGFN, and MKRA, achieved the best overall performance, with 88.2% precision, 82.1% recall, 85.0% F1-score, and 84.5% mAP50. Relative to the shared baseline, SASTE improved precision, recall, F1-score, and mAP50 by 5.4, 8.0, 6.8, and 10.5 percentage points, respectively, while increasing parameters by only 2.3 M and GFLOPs by 1.4 G. These results demonstrate that sparse attention, spatial-prior feed-forward modeling, and lightweight multi-kernel residual adaptation provide complementary gains in encoder representation.

### 3.3. Visualization Experiments

**Figure 10:**
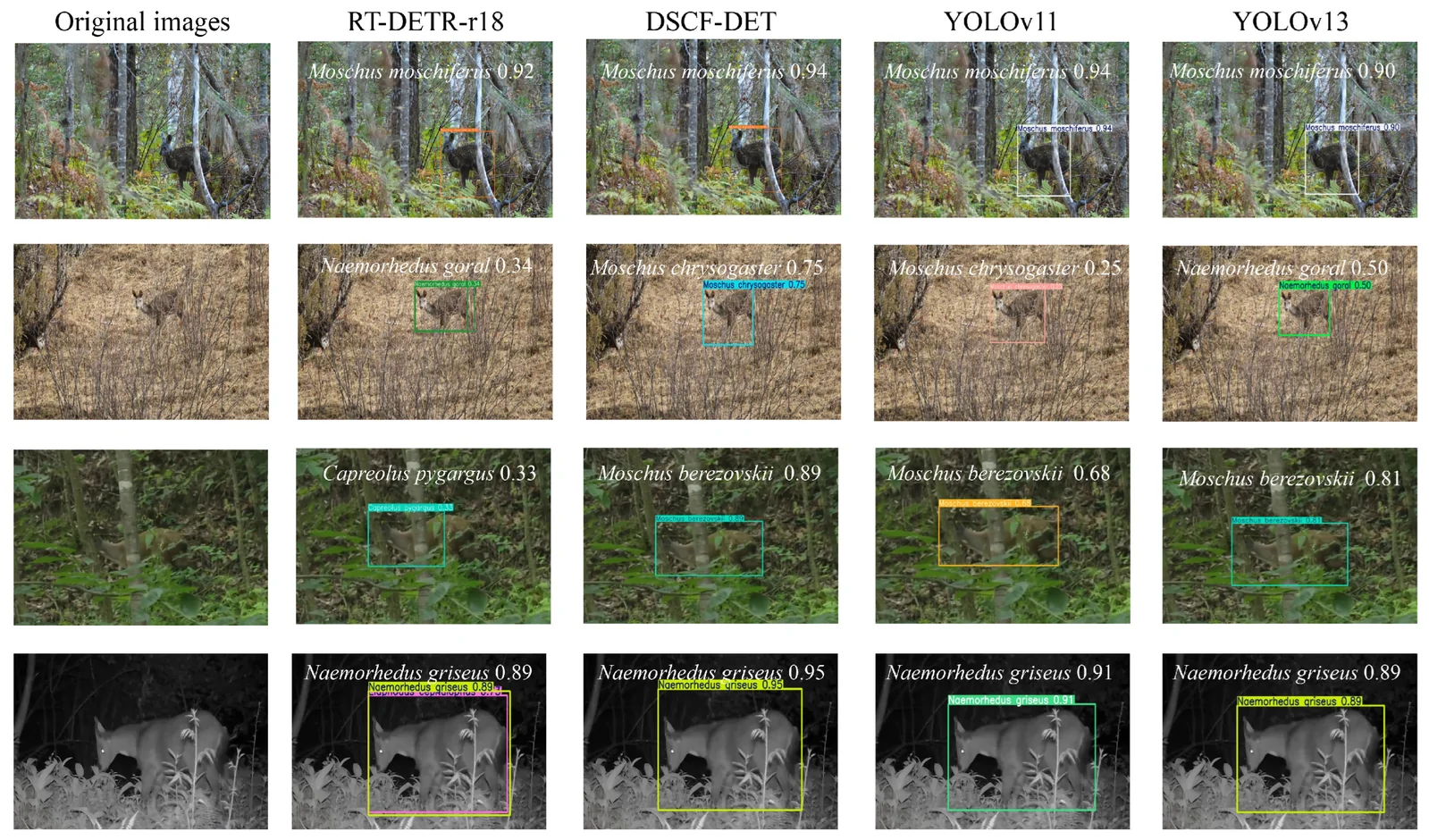
Visualization comparison of detection results under complex natural scenes. Bounding boxes and labels indicate the predicted target locations, species names, and confidence scores for each detector. Feature responses were further examined using heatmap visualizations and feature distribution maps (Figure 11 and Figure 12). Compared with RT-DETR, DSCF-DET generated more target-oriented activations around visible animal body regions, with reduced responses in irrelevant background areas such as vegetation, rocks, and shadows. This pattern suggests that the proposed modules improve foreground–background separation and strengthen the representation of discriminative animal features under camouflage and partial occlusion. These qualitative results are consistent with the quantitative improvements observed in the comparative and ablation experiments.

**Figure 11:**
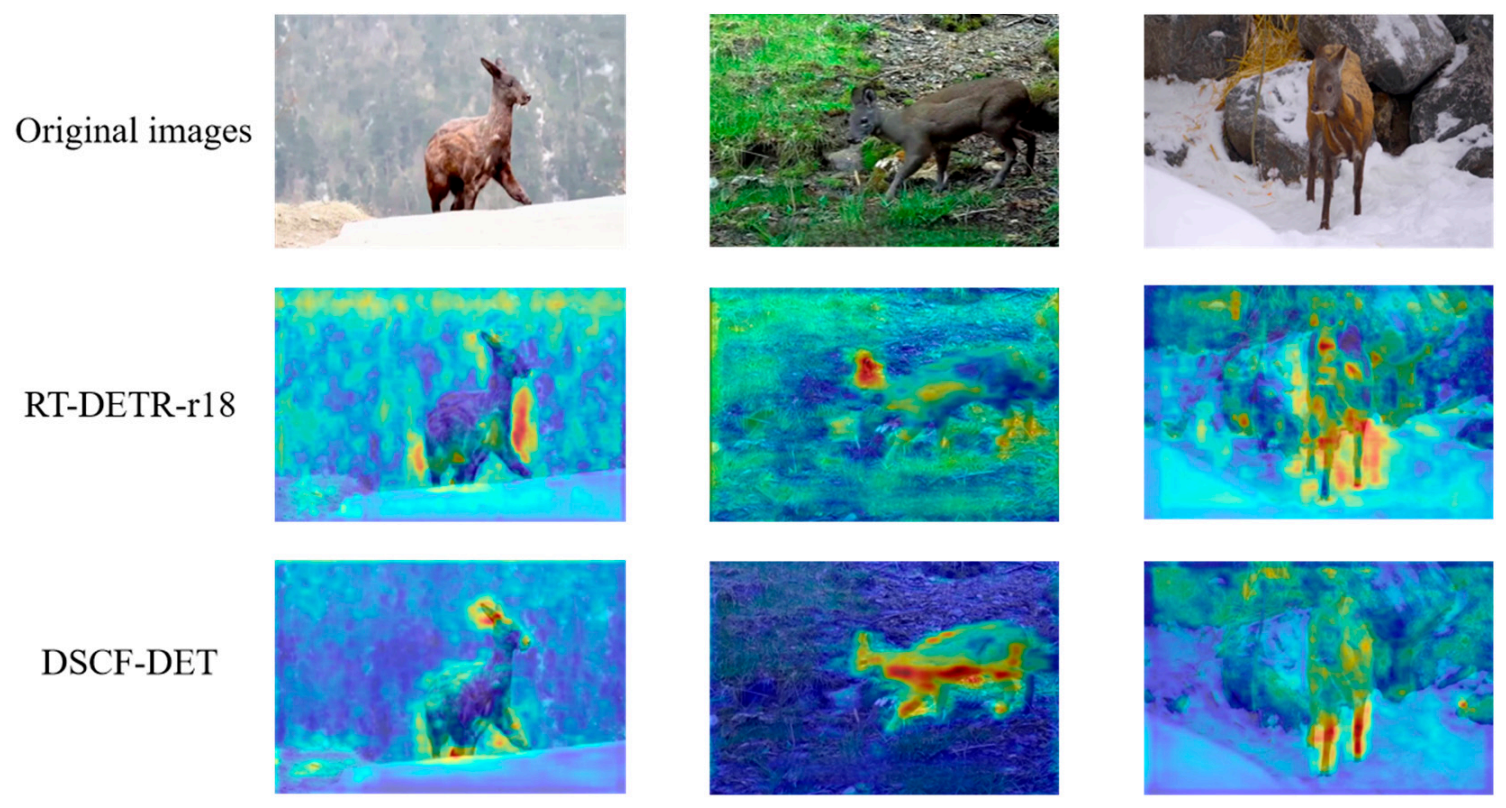
Heatmap visualization comparison between RT-DETR-r18 and DSCF-DET. The heatmaps are overlaid on representative original images; warmer colors indicate stronger response intensity, whereas cooler colors indicate weaker response intensity.

**Figure 12:**
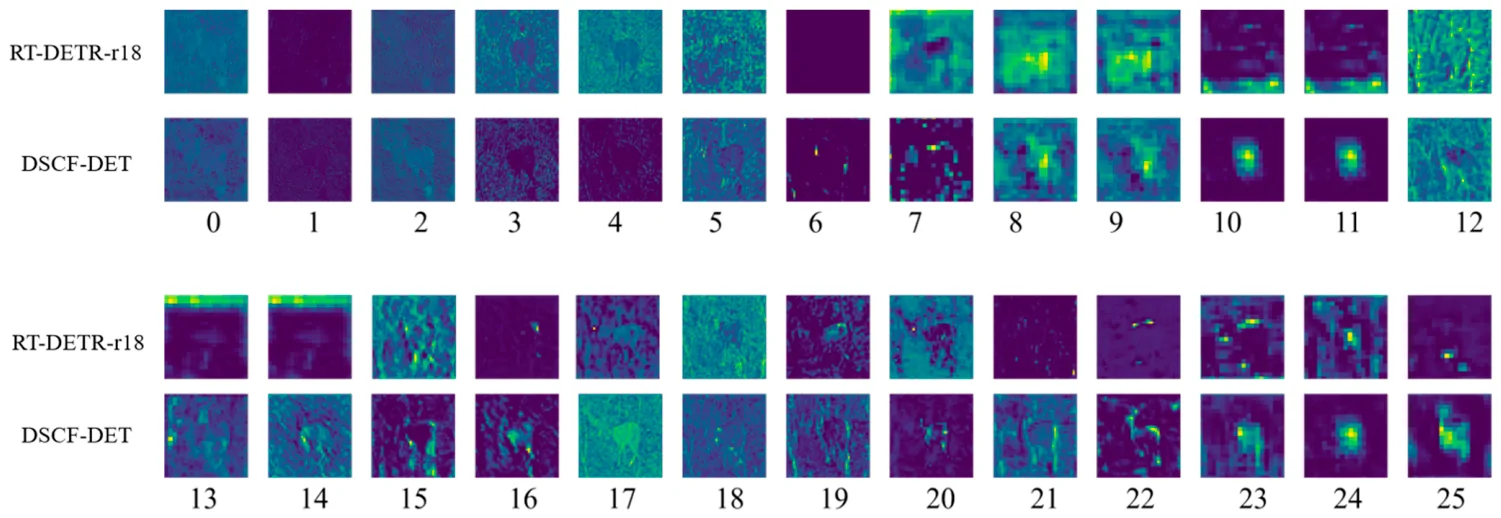
Feature activation map comparison between RT-DETR-r18 and DSCF-DET. The numbered columns (0–25) represent different feature channels. Color intensity indicates the normalized activation strength within each channel, with brighter regions corresponding to stronger activation responses.

To further examine the behavior of DSCF-DET in complex ecological scenes, qualitative visualizations were performed using representative images with camouflage, vegetation occlusion, cluttered backgrounds, and high inter-class similarity (Figure 10, Figure 11 and Figure 12).

Comparative detection results were evaluated in representative challenging scenes, including vegetation occlusion, background clutter, and nighttime imagery (Figure 10). The comparative models showed false detections and category confusion among visually similar artiodactyls. For example, YOLOv13 and RT-DETR-r18 misclassified *M. chrysogaster* as *N. goral*, whereas YOLOv11 produced the correct category but with a low confidence score of 0.25. In the detection of *M. berezovskii* and the nighttime *N. griseus* image, DSCF-DET maintained accurate localization and high-confidence predictions. These visual results indicate that DSCF-DET provides more stable localization and classification in complex wildlife images under both daytime and nighttime conditions.

### 3.4. Applicability Evaluation on an External Dataset

To further evaluate the applicability of the proposed model to an additional wildlife detection dataset, we conducted an experiment on the public wildlife detection dataset ENA24-detection [44]. ENA24-detection is a camera-trap-based wildlife object detection dataset. The public version used in this study contains 8789 images and 9772 bounding-box annotations, covering 22 common camera-trap target categories, including white-tailed deer, coyote, American black bear, raccoon, fox, opossum, squirrel, and wild turkey. This dataset is completely independent of the fine-grained musk deer dataset constructed in this study, and therefore provides a suitable external benchmark for evaluating model applicability under different species compositions, complex natural backgrounds, and independent acquisition conditions. In the experiment, ENA24-detection was converted into YOLO annotation format and divided into training, validation, and test sets using a fixed split ratio. Under the same data split and training settings, the baseline RT-DETR-r18 and the improved DSCF-DET were trained and evaluated to assess the effectiveness of the proposed architectural improvements on a public wildlife detection task.

Compared with RT-DETR-r18, DSCF-DET achieved slightly improved overall detection performance on the ENA24-detection dataset (Table 8). Specifically, precision increased from 93.2% to 95.0%, representing an improvement of 1.8 percentage points. The F1-score increased from 92.9% to 93.5%, and mAP50 improved from 93.9% to 94.4%, with a gain of 0.5 percentage points. Although recall decreased slightly from 92.6% to 92.1%, the change was limited, indicating that DSCF-DET maintained comparable recall while improving detection precision. Overall, these results indicate that DSCF-DET can maintain stable detection performance on an independent wildlife dataset after dataset-specific training, supporting its applicability to broader wildlife detection scenarios.

## 4. Discussion

This study addressed a fine-grained wildlife detection problem in which animal targets must be recognized under both complex ecological backgrounds and high inter-class similarity. In such scenes, vegetation occlusion, shadows, low visibility, and background camouflage can weaken target boundaries, while visually similar artiodactyls further increase the risk of category confusion. These challenges are consistent with previous camera-trap and wildlife-monitoring studies, in which complex backgrounds, incomplete body appearance, and morphologically similar species have been reported as important factors limiting detection reliability [23]. Therefore, the key difficulty in this study was not only to localize wildlife targets, but also to preserve discriminative visual cues for distinguishing musk deer from visually similar artiodactyls.

Previous YOLO-based wildlife detection studies have shown that improving feature extraction and adapting detectors to ecological backgrounds can effectively enhance detection performance [45,46,47]. The comparative results in this study support this view, as several YOLO-series detectors achieved competitive performance on the constructed fine-grained wildlife dataset. However, the present task places stronger emphasis on species-level discrimination under camouflage and occlusion. Accurate detection in this setting requires the model to capture subtle differences in body contour, fur texture, posture, and local appearance while suppressing interference from vegetation and cluttered backgrounds. This requirement provides the rationale for DSCF-DET, which integrates receptive-field-aware feature extraction, sparse spatial encoding, and cross-level feature fusion within the RT-DETR framework.

The comparison with mainstream detectors confirms that the proposed improvements substantially enhance the RT-DETR baseline. Compared with RT-DETR-r18, DSCF-DET achieved clear gains in precision, recall, F1-score, and mAP50, indicating that the redesigned framework improves both target localization and fine-grained discrimination. Among all compared models, DSCF-DET obtained the highest precision, which is particularly relevant for musk deer monitoring because false positives may cause species-level misidentification and increase manual verification costs. Although YOLOv5 and YOLOv8 showed strong performance in recall or mAP50, DSCF-DET maintained competitive overall accuracy with lower computational cost than several high-performing detectors, including YOLOv8, YOLOv13, RT-DETR-r34, and RT-DETR-r50. It also produced higher values than YOLOv26 across the four accuracy metrics. These results indicate that DSCF-DET provides a balanced solution for fine-grained wildlife detection, where reliable species discrimination and practical computational demand must be considered together.

Target size also influenced the relative performance of the evaluated detectors (Table S4). YOLOv5 achieved the highest mAP50 for small objects, whereas DSCF-DET outperformed YOLOv11 and YOLOv26 in this group and achieved higher recall and F1-score than YOLOv5, indicating that the proposed model also retained strong competitiveness for small targets. DSCF-DET further achieved the best performance for medium and large objects and consistently surpassed RT-DETR-r18 across all three size groups. The original DETR study similarly reported stronger performance for large objects but lower performance for small objects, partly attributing this pattern to the non-local computation of the Transformer architecture [21]. However, subsequent developments such as Deformable DETR demonstrated that small-object detection by transformer-based models can be substantially improved through multi-scale features and deformable attention [48]. More broadly, performance across object scales depends strongly on how spatial details and semantic information from different feature levels are preserved and integrated [22,49]. The present results therefore do not indicate a consistent division between YOLO- and transformer-based detectors. Instead, they suggest that scale-related performance is jointly influenced by the backbone, feature representation, and cross-scale fusion strategy. Taken together, the observed patterns should be viewed as relative trends within the present dataset rather than as fixed differences between detector families.

The ablation results further show that the performance gain of DSCF-DET comes from the complementary roles of the proposed modules. DRPBlock enhances receptive-field-aware feature extraction, which helps the backbone capture target-related local and contextual information in textured ecological scenes. This result is consistent with previous studies showing that adaptive receptive-field selection and deformable sampling can improve feature representation by better capturing objects with scale variation, geometric deformation, and complex spatial structures [50,51]. SASTE improves sparse spatial encoding by reducing redundant background interactions and strengthening informative spatial regions. This observation is also related to attention-based feature recalibration methods, in which channel and spatial attention are used to emphasize discriminative regions and suppress less informative responses [26]. CBAFusion contributes cross-level feature integration, allowing semantic and spatial information from different scales to be combined more effectively. Similar findings have been reported in feature-pyramid and bidirectional feature-fusion studies, where cross-scale aggregation improves multi-scale object representation and detection efficiency [30,49]. The complete model achieved the best overall performance, indicating that these components are not isolated additions but coordinated designs for addressing background interference and subtle inter-class differences in visually similar artiodactyls.

The visualization and cross-dataset experiments provide additional evidence for the practical value of DSCF-DET. The feature-response maps showed more concentrated activations on animal body regions and weaker responses to irrelevant background areas, indicating improved foreground–background separation in complex ecological images. However, this visualization analysis remains mainly qualitative, and model performance under extreme occlusion, severe illumination variation, or very small target sizes still requires further investigation. Moreover, when both RT-DETR-r18 and DSCF-DET were retrained and evaluated on the independent public ENA24-detection benchmark, DSCF-DET maintained a performance advantage over the baseline model. Because ENA24 differs in species composition, acquisition conditions, and ecological scenes, this result supports the applicability of DSCF-DET to another wildlife detection dataset under dataset-specific retraining settings.

Large-scale image-based wildlife monitoring often generates substantial image collections, making manual review a major component of the overall workflow. In nature reserves, national parks, and wildlife monitoring stations, DSCF-DET could be used as a preliminary screening tool for images obtained from camera traps and fixed monitoring devices. Images containing suspected musk deer or visually similar artiodactyls could be prioritized for verification by trained personnel, thereby retaining expert oversight while reducing the repetitive examination of empty, duplicate, or clearly nontarget images. This approach may be especially useful in remote mountainous and forest habitats, where repeated field surveys are difficult and image-based monitoring provides an important source of information. Recent studies have shown that AI-assisted workflows can reduce the manual review required for large camera-trap image collections and improve wildlife identification [15,52]. For musk deer conservation, more efficient screening could shorten the time required to identify target records and provide more timely information on species occurrence and activity, which may assist population monitoring and provide supporting information for habitat management and conservation planning.

As DSCF-DET operates on images acquired by existing camera-trap systems, no additional image-acquisition equipment is required for deployment. Instead, images retrieved from field devices could be processed centrally or offline at monitoring stations and research institutions, avoiding the need to equip every camera with high-performance computing hardware. This deployment strategy minimizes additional hardware investment by relying primarily on centralized computing resources and existing monitoring infrastructure. The principal operational benefit of DSCF-DET lies in reducing the number of images requiring full manual inspection, thereby lowering labor requirements and associated image-review costs while preserving expert confirmation for uncertain or conservation-relevant records. A field-based comparison of camera-trap monitoring workflows found that AI-assisted image classification reduced processing costs, with further savings achieved when automated classification was combined with network-connected cameras [53]. Incorporating DSCF-DET as a software-based screening component may therefore provide a practical and potentially economically feasible complement to conventional image review, particularly for long-term monitoring programs that produce large and continuously expanding image collections. Nevertheless, the actual cost-effectiveness of DSCF-DET will depend on the scale of deployment, available computing resources, and local monitoring workflows, and should be evaluated prospectively under operational field conditions.

Although DSCF-DET showed consistent improvements over the RT-DETR baseline in the comparative and ablation experiments, the reported performance should be interpreted in relation to the training configuration adopted in this study. Hyperparameters such as learning rate, batch size, number of training epochs, optimization schedule, regularization settings, input resolution, and augmentation configuration can affect model convergence and final detection performance. Applying identical training settings across all detectors reduced procedural differences and provided a controlled basis for comparison. The influence of hyperparameter configuration has also been illustrated in previous empirical studies. Wang et al. incorporated genetic algorithm-based parameter optimization into an SVR prediction framework, showing that parameter-selection strategy is an important part of model development [54]. Hwang et al. further showed that image-augmentation effects were configuration-dependent in object detection and instance segmentation, with different augmentation strategies producing different effects on model accuracy [55]. These findings support the need to consider parameter and augmentation settings when interpreting comparative model performance. The backbone comparison in this study provides a more tightly controlled setting, as only the feature-extraction network was replaced within the same RT-DETR framework while the remaining detector components and experimental settings were kept unchanged. Architecture-specific hyperparameter optimization may further improve the performance of individual detectors. Therefore, future comparisons could examine hyperparameter sensitivity under comparable search spaces, optimization criteria, and computational budgets.

Stochastic processes are also relevant to deep learning-based model comparison. A random seed can affect dataset shuffling or partitioning, initial model weights, mini-batch ordering, and random augmentation sampling. These processes influence the optimization trajectory of stochastic gradient-based training and may lead to different learned representations even when the model architecture and nominal training settings remain unchanged. Bouthillier et al. reported that variance arising from data sampling, parameter initialization, augmentation, and hyperparameter choices can markedly affect machine-learning benchmarks [56]. Picard further showed that random-seed selection can produce measurable performance variation in common computer-vision architectures [57]. These studies indicate that random factors are part of the experimental context for detector comparison and should be considered together with architecture design, implementation source, and training protocol.

Despite these advantages, several aspects remain important for future improvement. The self-constructed dataset includes complex backgrounds and visually similar artiodactyls, but rare poses, seasonal changes, extreme illumination, and long-term camera-trap image streams may still be underrepresented, which is a common challenge in wildlife image datasets [58]. In addition, although the ENA24 experiment provides useful cross-dataset evidence, real field monitoring may involve greater variation in camera devices, installation angles, habitats, and animal movement patterns. Future work should therefore extend DSCF-DET to more diverse taxa, ecological scenes, and continuous monitoring settings. Further optimization through model compression, edge-device inference, and video-based wildlife monitoring may also improve its value for long-term intelligent ecological monitoring systems [59,60].

## 5. Conclusions

This study proposed DSCF-DET, an RT-DETR-based framework for fine-grained detection of musk deer and visually similar artiodactyls in complex natural scenes. By integrating DRPBlock, SASTE, and CBAFusion, the proposed framework improved receptive-field-aware feature extraction, sparse spatial encoding, and cross-level feature fusion. These designs enhanced the representation of wildlife targets affected by camouflage, partial occlusion, and high inter-class similarity.

Experimental results on the constructed fine-grained wildlife dataset showed that DSCF-DET achieved 91.4% precision, 86.2% recall, 88.7% F1-score, and 86.4% mAP50. Compared with the baseline RT-DETR-r18, DSCF-DET improved precision, recall, F1-score, and mAP50 by 8.6, 12.1, 10.5, and 12.4 percentage points, respectively, while maintaining moderate model complexity with 23.7 M parameters and 64.4 GFLOPs. Visualization results further indicated that DSCF-DET produced more target-focused feature responses and improved foreground–background separation under complex ecological conditions.

Overall, this study provides a task-oriented and computationally balanced detection framework for fine-grained ecological image analysis and intelligent wildlife monitoring.

## Figures and Tables

**Table 1:** Statistical distribution of the constructed fine-grained wildlife dataset. Values indicate the number of images.

Category	Original Images	Train	Val	Test
*Moschus moschiferus*	283	204	23	56
*Moschus berezovskii*	173	121	17	35
*Moschus chrysogaster*	190	133	19	38
*Moschus anhuiensis*	62	43	6	13
*Moschus fuscus*	52	36	5	11
*Elaphodus cephalophus*	169	118	17	34
*Hydropotes inermis*	219	153	22	44
*Capreolus pygargus*	271	189	27	55
*Naemorhedus goral*	105	73	11	21
*Naemorhedus griseus*	159	111	16	32
Total	1683	1181	163	339

**Table 2:** Experimental environment used for model training and evaluation.

Experimental Environment	Parameter or Version
Operating system	Ubuntu 22.04
GPU	NVIDIA GeForce RTX 3090, 24 GB
Python	3.10.13
PyTorch	2.1.0
CUDA	12.1
cuDNN	8.9.2

**Table 3:** Performance comparison of different object detection models on the fine-grained wildlife dataset.

Model	Precision (%)	Recall (%)	F1-Score (%)	mAP50 (%)	Params (M)	GFLOPs (G)
YOLOv5m	89.2	90.5	89.8	87.3	25.0	64.0
YOLOv6s	75.0	71.3	73.1	75.8	16.2	44.0
YOLOv8m	88.9	80.1	84.3	88.3	25.8	78.7
YOLOv9m	82.4	70.7	76.1	79.6	20.0	76.5
YOLOv11m	84.8	76.2	80.3	84.1	20.0	67.7
YOLOv13l	85.2	81.1	83.1	87.1	27.5	88.1
YOLOv26m	70.0	71.2	70.6	76.3	21.7	67.9
RT-DETR-r18	82.8	74.1	78.2	74.0	19.9	57.0
RT-DETR-r34	86.1	76.4	81.0	78.8	31.1	88.8
RT-DETR-r50	85.9	74.4	79.7	78.3	41.9	129.6
Faster R-CNN	48.6	63.9	55.2	58.3	137.1	370.2
DSCF-DET	91.4	86.2	88.7	86.4	23.7	64.4

Note: Baseline model references include Faster R-CNN [20], YOLOv5 [31], YOLOv6 [32], YOLOv8 [33], YOLOv9 [34], RT-DETR [22], YOLOv11 [35], YOLOv13 [36] and YOLOv26 [37].

**Table 4:** Performance comparison of different backbone networks.

Backbone	Precision (%)	Recall (%)	F1-Score (%)	mAP50 (%)	Params (M)	GFLOPs (G)
ResNet-18	82.8	74.1	78.2	74.0	19.9	57.0
MobileNetV4	82.3	71.4	76.5	72.7	11.3	39.5
EfficientViT	86.6	75.5	80.7	77.2	10.7	27.3
OverLock	87.1	77.8	82.2	82.2	25.5	62.1
ConvNext-V2	81.9	72.0	76.6	72.9	12.3	31.9
MambaOut	86.1	71.4	78.1	75.3	15.9	41.9
DRPBlock	89.6	82.9	86.1	83.2	17.5	50.3

Note: Baseline backbone references include ResNet-18 [38], MobileNetV4 [39], EfficientViT [40], OverLock [41], ConvNeXt-V2 [42], and MambaOut [43].

**Table 5:** Ablation results of the proposed modules.

Model	A	B	C	Precision (%)	Recall (%)	F1-Score (%)	mAP50 (%)	Params (M)	GFLOPs (G)
1	-	-	-	82.8	74.1	78.2	74.0	19.9	57.0
2	√	-	-	89.6	82.9	86.1	83.2	17.5	50.3
3	-	√	-	88.2	82.1	85.0	84.5	22.2	58.4
4	-	-	√	87.5	81.1	84.2	84.7	23.7	69.8
5	√	√	-	90.7	83.6	87.0	86.2	19.9	51.7
6	√	-	√	88.2	81.8	84.9	82.0	21.3	63.0
7	-	√	√	87.4	79.5	83.3	83.3	26.0	71.2
8	√	√	√	91.4	86.2	88.7	86.4	23.7	64.4

Note: “√” denotes the inclusion of the corresponding module, whereas “-” denotes its absence.

**Table 6:** Ablation results of DRPBlock.

Model	$F_{loc}$	$F_{sur}$	CCSRUnit	Precision (%)	Recall (%)	F1-Score (%)	mAP50 (%)	Params (M)	GFLOPs (G)
1	-	-	-	82.8	74.1	78.2	74.0	19.9	57.0
2	√	-	-	85.7	78.6	82.0	80.7	13.6	42.0
3	√	√	-	86.6	80.0	83.2	81.7	15.0	45.5
4	√	√	√	89.6	82.9	86.1	83.2	17.5	50.3

Note: “√” denotes the inclusion of the corresponding module, whereas “-” denotes its absence.

**Table 7:** Ablation results of SASTE. The baseline encoder (Model 1) uses standard multi-head self-attention (MHSA) and a feed-forward network (FFN). WGSSA, SPGFN, and MKRA are progressively introduced to form SASTE.

Model	WGSSA	SPGFN	MKRA	Precision (%)	Recall (%)	F1-Score (%)	mAP50 (%)	Params (M)	GFLOPs (G)
1	-	-	-	82.8	74.1	78.2	74.0	19.9	57.0
2	√	-	-	83.9	74.2	78.8	78.2	20.7	57.9
3	√	√	-	86.7	78.7	82.5	81.7	22.1	58.3
4	√	√	√	88.2	82.1	85.0	84.5	22.2	58.4

Note: “√” denotes the inclusion of the corresponding module, whereas “-” denotes its absence.

**Table 8:** Performance comparison of RT-DETR-r18 and DSCF-DET on the public ENA24-detection benchmark.

Model	Precision (%)	Recall (%)	F1-Score (%)	mAP50 (%)
RT-DETR-r18	93.2	92.6	92.9	93.9
DSCF-DET	95.0	92.1	93.5	94.4

## Data Availability

The dataset generated and analyzed in this study is available in Zenodo at https://doi.org/10.5281/zenodo.21373730.

## Supplementary Materials

The following supporting information can be downloaded at: https://www.mdpi.com/article/10.3390/biology15161344/s1, Figure S1. Target box size distribution of annotated objects. Figure S2. Distribution of original image resolutions. MP denotes megapixels, calculated as image width × image height/1,000,000. Table S1. Number of annotated objects per species in the constructed fine-grained wildlife dataset. Table S2. Summary of image-level attributes in the fine-grained wildlife dataset. Table S3. Comparison of RT-DETR-r18 performance with and without data augmentation. Table S4. Object-size-based detection performance of representative models on the test set.
