# Supplementary material for: DSCF-DET: An RT-DETR-Based Framework for Fine-Grained Detection of Musk Deer and Visually Similar Artiodactyls
Source: Biology (Basel). 2026 Aug 8;15(16):1344. doi: 10.3390/biology15161344 (PMC13509854; doi:10.3390/biology15161344)
Supplement: Supplementary file 1 [file biology-15-01344-s001.zip › biology-4450019-supplementary.pdf]

Supplementary material

Table S1. Number of annotated objects per species in the constructed fine-grained wildlife dataset.

Category	Original objects	Train objects	Val objects	Test objects
Moschus moschiferus	317	232	27	58
Moschus berezovskii	189	133	19	37
Moschus chrysogaster	202	141	23	38
Moschus anhuiensis	63	43	7	13
Moschus fuscus	55	37	6	12
Elaphodus cephalophus	203	140	21	42
Hydropotes inermis	239	165	27	47
Capreolus pygargus	343	237	39	67
Naemorhedus goral	119	83	12	24
Naemorhedus griseus	181	125	18	38
Total	1911	1336	199	376

To characterize variation in target scale, the width and height of each annotated bounding box were normalized by the corresponding image dimensions. The resulting distribution reflects the relative sizes and aspect ratios of wildlife targets across the dataset.

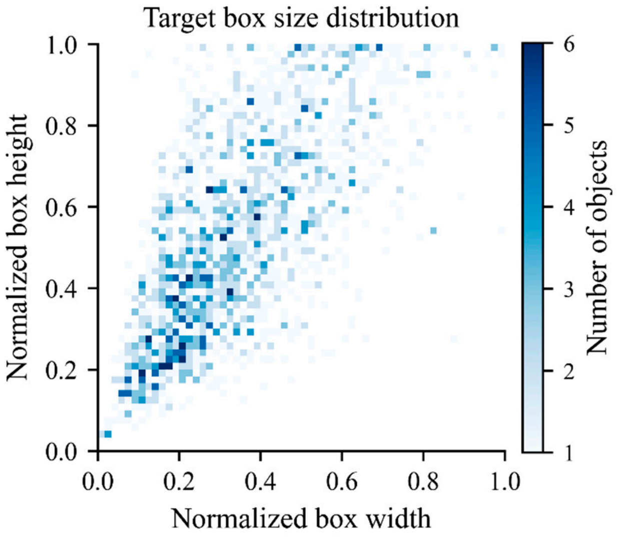

Figure S1. Target box size distribution of annotated objects. The width and height values were normalized by image size, and darker colors indicate a higher number of annotated objects.

Because the images were collected from multiple ecological image sources, their original resolutions varied considerably, ranging from 275 × 229 to 5760 × 4964 pixels. The distribution of original image resolutions was summarized in megapixels to characterize this heterogeneity.

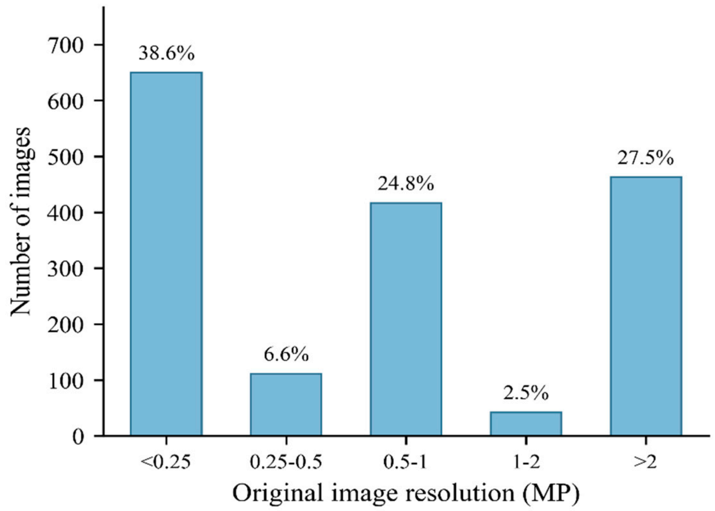

Figure S2. Distribution of original image resolutions. MP denotes megapixels, calculated as image width × image height / 1,000,000.

We summarized the image-level proportions of daytime, nighttime/twilight, occluded, and camouflaged samples across the whole dataset. Daytime and nighttime/twilight were treated as mutually exclusive illumination categories, whereas occlusion and camouflage were treated as independent image attributes. Therefore, occluded and camouflaged samples could overlap with each other and could occur under either daytime or nighttime/twilight conditions.

Table S2. Summary of image-level attributes in the fine-grained wildlife dataset.

Category	Count	Proportion (%)
daytime	1559	92.6
nighttime	124	7.4
occluded	1077	64.0
camouflaged	943	56.0

Effect of the data augmentation strategy

Table S3. Comparison of RT-DETR-r18 performance with and without data augmentation.

Model	Precision(%)	Recall(%)	F1-score (%)	mAP50 (%)
RT-DETR-r18 (without data augmentation)	78.5	**74.9**	76.7	72.9
RT-DETR-r18 (with data augmentation)	**82.8**	74.1	**78.2**	**74.0**

Note: Both models were evaluated on the same test set using identical evaluation settings. The comparison represents the combined effect of the complete augmentation pipeline rather than the independent contribution of any individual transformation.

RT-DETR-R18 was trained under two conditions, with and without the complete data augmentation pipeline described in the manuscript, and both models were evaluated on the same test set using identical evaluation settings. The augmented model achieved higher precision, F1-score, and mAP50, while recall decreased slightly from 74.9% to 74.1% (Table S3). Specifically, precision increased from 78.5% to 82.8%, the F1-score increased from 76.7% to 78.2%, and mAP50 increased from 72.9% to 74.0%. These

results indicate that the complete augmentation pipeline improved the overall detection performance of RT-DETR-R18 on the constructed wildlife dataset. Because the pipeline included multiple geometric, photometric, weather-related, and illumination-related transformations, the observed improvement should be interpreted as their combined effect rather than as evidence for the independent contribution of vertical flipping or any other single operation.

Object-Size-Based Performance Analysis

An object-size-based evaluation was further conducted to examine whether detection performance differed across apparent target scales. In this analysis, object size refers to the relative size of the annotated target in the image, rather than the actual body size of the animal. This distinction is important for wildlife images, because the same or similar species may appear at markedly different scales depending on camera distance, animal posture, occlusion, and image composition.The analysis was performed on the original test set using the trained weights from the main experiments. The original training, validation, and test splits were not changed. For each annotated object, the normalized bounding-box area was calculated as the product of the normalized box width and height in the YOLO-format annotation. The test-set objects were then divided into small, medium, and large groups according to the tertiles of the bounding-box area distribution. This supplementary analysis was intended to reveal scale-related performance patterns among representative detectors. It should therefore be interpreted as an additional diagnostic analysis rather than a replacement for the overall test-set evaluation reported in the main manuscript.

Table S4. Object-size-based detection performance of representative models on the test set.

Model	Object size	Precision (%)	Recall (%)	F1-score (%)	mAP50 (%)
YOLOv5	S	**80.2**	71.9	75.8	**69.0**
	M	**89.3**	85.9	87.6	85.1
	L	79.4	74.1	76.7	72.2
YOLOv11	S	71.1	68.9	70.0	62.0
	M	89.1	81.1	84.9	78.6
	L	76.7	69.5	72.9	63.6
YOLOv26	S	78.0	55.9	65.1	54.4
	M	80.0	67.6	73.3	62.5
	L	74.2	56.3	64.0	53.9
RT-DETR-r18	S	73.8	66.1	69.7	57.6
	M	85.6	76.7	80.9	74.7
	L	74.2	70.4	72.3	63.7
DSCF-DET	S	79.3	**76.1**	**77.7**	67.4
	M	87.4	**89.8**	**88.6**	**86.2**
	L	**87.1**	**77.1**	**81.8**	**76.0**

Note: S, M, and L denote small, medium, and large annotated objects, respectively. Objects were grouped according to tertiles of normalized bounding-box area in the original test set. The original data split and trained model weights were kept unchanged.

The object-size-based results showed that model performance varied across apparent target scales. Among the YOLO-series detectors, YOLOv5 showed the most stable performance across the three object-size groups, with strong results for both small and medium objects. YOLOv11 performed well for medium objects but showed a more evident decrease for large objects, whereas YOLOv26 showed relatively lower performance across all three groups. For the RT-DETR-based detectors, DSCF-DET

consistently outperformed the RT-DETR-r18 baseline in small, medium, and large object groups, indicating that the proposed modifications improved the scale-related detection ability of the baseline framework. DSCF-DET achieved the strongest performance for medium objects and remained competitive for large objects. These results suggest that object-size-related performance was not determined only by whether a detector was YOLO-based or transformer-based, but also depended on the specific architecture and feature enhancement strategy of each model.
